# Fluorescence‐Quenching Lateral Flow Immunoassay for “Turn‐On” and Sensitive Detection of Anti‐SARS‐Cov‐2 Neutralizing Antibodies in Human Serum

**DOI:** 10.1002/advs.202305774

**Published:** 2023-11-30

**Authors:** Lun Bian, Qiangqiang Fu, Zhuoheng Gan, Ze Wu, Yuchen Song, Yufeng Xiong, Fang Hu, Lei Zheng

**Affiliations:** ^1^ Biomaterials Research Center School of Biomedical Engineering Southern Medical University Guangzhou 510515 China; ^2^ Department of Laboratory Medicine Nanfang Hospital Southern Medical University Guangzhou 510515 China; ^3^ Division of Laboratory Medicine Zhujiang Hospital Southern Medical University Guangzhou 510282 China

**Keywords:** aggregation‐induced emission, fluorescence‐quenching, lateral flow immunoassay, neutralizing antibodies, SARS‐CoV‐2

## Abstract

The titer of anti‐severe acute respiratory syndrome coronavirus 2 (SARS‐CoV‐2) neutralizing antibodies (NAbs) in the human body is an essential reference for evaluating the acquired protective immunity and resistance to SARS‐CoV‐2 infection. In this study, a fluorescence‐quenching lateral flow immunoassay (FQ‐LFIA) is established for quantitative detection of anti‐SARS‐CoV‐2 NAbs in the sera of individuals who are vaccinated or infected within 10 min. The ultrabright aggregation‐induced emission properties encapsulated in nanoparticles, AIE_490_NP, are applied in the established FQ‐LFIA with gold nanoparticles to achieve a fluorescence “turn‐on” competitive immunoassay. Under optimized conditions, the FQ‐LFIA quantitatively detected 103 positive and 50 negative human serum samples with a limit of detection (LoD) of 1.29 IU mL^−1^. A strong correlation is present with the conventional pseudovirus‐based virus neutralization test (*R* ^*2*^ = 0.9796, *P* < 0.0001). In contrast, the traditional LFIA with a “turn‐off” mode can only achieve a LoD of 11.06 IU mL^−1^. The FQ‐LFIA showed excellent sensitivity to anti‐SARS‐CoV‐2 NAbs. The intra‐ and inter‐assay precisions of the established method are below 15%. The established FQ‐LFIA has promising potential as a rapid and quantitative method for detecting anti‐SARS‐CoV‐2 NAbs. FQ‐LFIA can also be used to detect various biomarkers.

## Graphical abstract

A quantitative detection LFIA method for anti‐SARS‐CoV‐2 NAbs is developed based on the ability of NAbs to compete with ACE2 for binding to the RBD. Using the FRET effect between AuNPs and AIE nanoparticles (AIE_490_NP), the competition method distinguishes results from conventional signals that weaken with increasing concentration but reflect the results in a “turn‐on” manner.

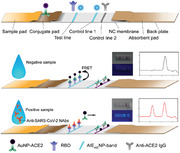

## 1 Introduction

Severe acute respiratory syndrome coronavirus 2 (SARS‐CoV‐2) has caused the global pandemic coronavirus disease 2019 (COVID‐19) that has killed millions worldwide.^[^ ^1^, ^2^ ^]^ Fortunately, COVID‐19 has been controlled in many countries through widespread vaccination and herd immunization strategies.^[^ ^3^ ^]^ These strategies provide individuals who are vaccinated or infected with high titers of anti‐SARS‐CoV‐2 neutralizing antibodies (NAbs),^[^ ^4^ ^]^ which can neutralize SARS‐CoV‐2 and inhibit its binding to angiotensin‐converting enzyme 2 (ACE2) in human cell membranes.^[^ ^5^ ^]^ Detecting anti‐SARS‐CoV‐2 NAbs titers in serum can effectively evaluate human resistance to SARS‐CoV‐2 infection and guide further vaccine strategies.^[^ ^6^ ^]^

Since the SARS‐CoV‐2 relies on the receptor‐binding domain (RBD) in the spike glycoprotein to bind with ACE2, the titer of anti‐SARS‐CoV‐2 NAbs in human serum was detected mainly by evaluating its capacity to inhibit the combination of RBD and ACE2.^[^ ^7^ ^]^ However, routine methods such as the conventional virus neutralization test (VNT) and pseudovirus‐based VNT (pVNT) require biosafety level 3 or level 2 facilities,^[^ ^8^, ^9^ ^]^ respectively, owing to the demand for active virus operations or cell culturing. The strict experimental condition requirements make VNT and pVNT difficult to generalize in primary hospitals and substantially less applicable to self‐monitoring in the general population.^[^ ^10^ ^]^ Recently, several immunoassays have been established for detecting anti‐SARS‐CoV‐2 NAbs, offering easy operation and simple condition requirements, including enzyme‐linked immunosorbent assay (ELISA),^[^ ^11^ ^]^ surface plasmon resonance assay (SPR),^[^ ^12^ ^]^ digital microfluidic assay,^[^ ^13^ ^]^ and lateral flow immunoassay (LFIA).^[^ ^14^ ^]^ Among these methods, LFIA has attracted widespread attention and rapid development in recent years owing to its rapidity, simplicity, and cost.^[^ ^15^ ^]^ Labels for LFIA have been developed from gold nanoparticles (AuNPs) and colored latex nanoparticles^[^ ^16^, ^17^ ^]^ only applied for qualitative detection, to the currently commonly used fluorescent nanoparticles which can achieve quantitative detection with the assistance of fluorescence data obtained using a portable fluorescence reader.^[^ ^18^ ^]^

The performance of fluorescent nanoparticle‐based LFIA, including accuracy and sensitivity, highly depends on the fluorescence label employed.^[^ ^19^ ^]^ Therefore, finding bright and economical fluorescent labels for LFIA is indispensable to LFIA research. Recently, organic luminogens with aggregation‐induced emission properties (AIEgens) have been encapsulated in polystyrene (PS) nanoparticles for use in LFIA, with satisfactory progress.^[^ ^14^, ^20^, ^21^ ^]^ AIEgens encapsulated in PS nanoparticles produce ultrabright fluorescence owing to the restriction of intermolecular motion in the aggregated state and the restriction of molecular motion from benzene rings in styrene.^[^ ^22^ ^]^ Unlike the traditional quantum dot nanoparticles and lanthanide‐chelated nanoparticles commonly used in LFIA, AIEgen‐based nanoparticles (AIENP) have the advantages of tuned fluorescence wavelength, ultra‐brightness, and economic cost,^[^ ^23^ ^]^ which are essential in practical applications. However, LFIA with a competitive strategy often suffers from low sensitivity as it requires sufficient analytes to reduce the signals on the test (T) line (signal “turn off”). In the fluorescence‐quenching LFIA (FQ‐LFIA), the fluorescent material is sprayed on the T line, the fluorescence‐quenching material competes with the analytes, and the fluorescent signal on the T line is positively correlative to the analyte concentration to supply a “turn‐on” mode, theoretically improving the sensitivity.

**Scheme 1:**
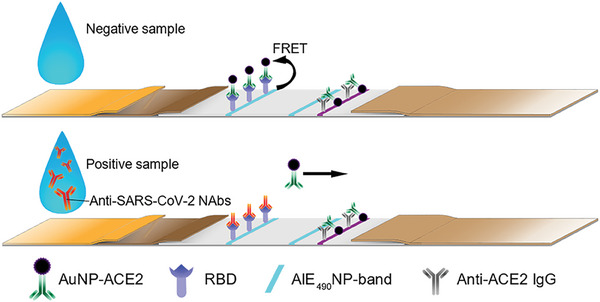
Schematic of the established FQ‐LFIA test strip for detecting anti‐SARS‐CoV‐2 NAbs in human serum samples.

In this study, we established an FQ‐LFIA using bright AIENP as a fluorescent material and AuNPs as a fluorescence‐quenching material, enabling quantitative detection of anti‐SARS‐CoV‐2 NAbs in human serum. An AIEgen with bright green fluorescence, AIE_490_, was encapsulated into carboxyl‐modified PS nanoparticles to obtain ultrabright AIEgen‐based PS nanoparticles (AIE_490_NP), which were coated on a nitrocellulose (NC) membrane as the T line. AuNPs were modified with an ACE2 Fc chimera (AuNP‐ACE2) and stabilized on a conjugate pad as a fluorescence‐quenching marker. When testing negative samples (without NAbs), the AuNP‐ACE2 on the T line due to ACE2‐RBD recognition quenched the fluorescence signal through the fluorescence resonance energy transfer (FRET) effect, resulting in dim fluorescence. In contrast, when testing positive samples, the NAbs prevented the AuNP‐ACE2 and RBD from combining, and the T line exhibited an increase in fluorescence positively correlated with the NAbs titer (**Scheme** **1**). The SARS‐CoV‐2 NAbs in serum samples (Table S1, Supporting Information) can be quantitively detected, and the limit of detection (LoD) reaches 1.29 IU mL^−1^, substantially more sensitive than conventional LFIA (LoD = 11.06 IU mL^−1^) and commercial ELISA (LoD = 100.39 IU mL^−1^), taking advantage of the fluorescence‐quenching strategy and “turn‐on” mode.

## 2 Results

### 2.1 Synthesis and Characterization of Conjugated AIE490NP

**Figure 1:**
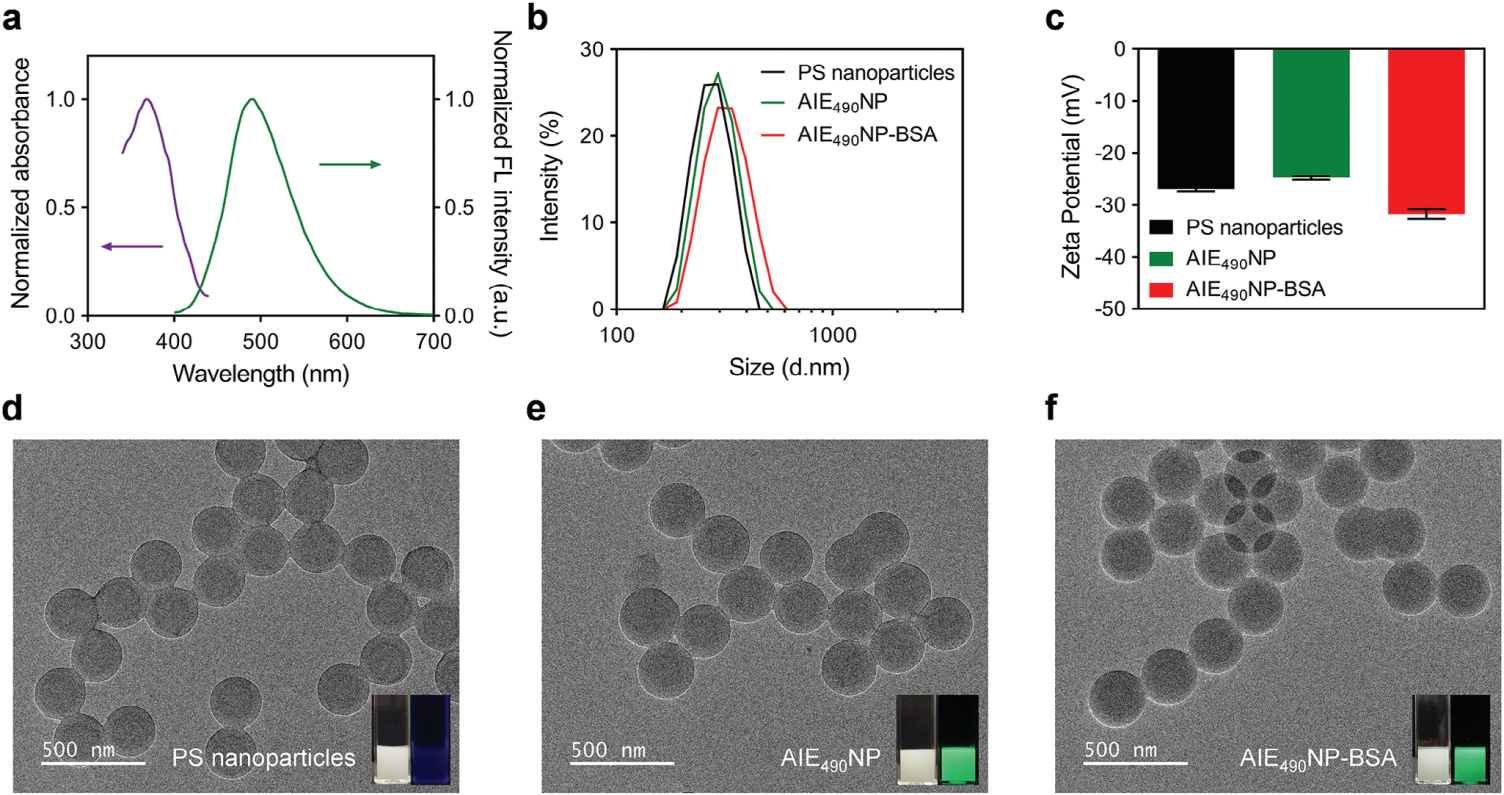
a) The absorption and fluorescence spectra of AIE_490_NP. The hydrodynamic diameter b) and zeta potential c) of PS nanoparticles, AIE_490_NP, and AIE_490_NP‐BSA. All particles were diluted in water to 100 µg mL^−1^. The TEM images of PS nanoparticles d), AIE_490_NP e), and AIE_490_NP‐BSA f); the insets show PS nanoparticles, AIE_490_NP, and AIE_490_NP‐BSA under white light (left) and UV light (right, *λ*_ex_ = 365 nm).

AIE_490_ is a blue‐emissive AIEgen, as reported in our previous work.^[^ ^14^ ^]^ As shown in **Figure** **1**a, the absorbance and fluorescence of AIE_490_ aggregated in water (THF/water, v/v = 1/99, 10 µg mL^−1^) have maximum values at 359 and 495 nm, respectively (Figure S1, Supporting Information). The AIE properties of AIE_490_ were examined by monitoring the fluorescence intensity of 100 µg AIE_490_ dissolved in THF/water mixtures with different THF proportions. The fluorescence intensity of AIE_490_ increased gradually as the water fraction increased from 0% to 80%, and increased significantly from 80% to 99%, indicating the AIE properties of AIE_490_ (Figure S2, Supporting Information).

The fluorescence stability of AIE_490_ was tested by monitoring the fluorescence intensity trends of AIE_490_ dissolved in water (THF/water, v/v = 1/99, 10 µg mL^−1^) under continuous irradiation of white light (100 mW cm^−2^) or different storage temperatures (10, 20, 30, 40, and 50 °C) and pH levels (pH 5.5, pH 6.8, pH 7.4, pH 8, and pH 9). The fluorescence intensity of AIE_490_ exhibited only an 8.0% loss under 60 min of white‐light irradiation (Figure S3, Supporting Information), a maximum loss of 6.1% under different temperatures (Figure S4 and Table S3, Supporting Information), and an 8.3% loss under different pH levels (Figure S5 and Table S4, Supporting Information), demonstrating satisfactory fluorescence stability for further detection.

For application in FQ‐LFIA, AIE_490_ molecules were encapsulated in 300 nm carboxyl‐modified PS nanoparticles using the swelling method to obtain AIE_490_NP. The photoluminescence quantum yields (PLQYs) of AIE_490_ aggregated in water and AIE_490_NP were 31.4% and 33.8%, respectively. The number of AIE_490_ molecules encapsulated in an AIE_490_NP was calculated to be 1.01 × 10^6^ (Figure S6, Supporting Information). The fluorescence intensity of AIE_490_NP is 10.314 times that of AIE_490_ aggregated in water when the concentration of the AIE_490_ molecule is the same at 0.897 µg mL^−1^ (Figure S7, Supporting Information). The maximum absorption and fluorescence of AIE_490_NP were observed at 368 and 490 nm (Figure 1a), respectively, similar to those of AIE_490_. The AIE_490_NP also exhibited excellent fluorescence stability against white light irradiation (3.0% loss, Figure S3, Supporting Information), temperature (2.3% loss, Figure S8, and Table S3, Supporting Information), and pH (4.9% loss, Figure S9, and Table S4, Supporting Information). Meanwhile, the structural stability of AIE_490_NP was revealed by monitoring the hydrodynamic diameter of AIE_490_NP stored at different temperatures (291.9–314.4 nm, Figure S10, Supporting Information) and pH levels (291.2–313.4 nm, Figure S11, Supporting Information). To ensure the capability of the AIE_490_NP coating on the NC membrane, BSA was conjugated onto the surface of AIE_490_NP using the EDC/NHS method. The hydrodynamic diameter (Figure 1b) and zeta potential (Figure 1c) of AIE_490_NP were 287.8 nm and−26.9 mV, similar to those of PS nanoparticles (269.8 nm and−24.8 mV), while those of AIE_490_NP‐BSA were 305.0 nm and −31.7 mV, which of the change was caused mainly by the modification of protein on the surface. Transmission electron microscopy (TEM) images (Figure 1d–f) of PS nanoparticles, AIE_490_NP, and AIE_490_NP‐BSA also confirmed that the encapsulation of AIE_490_ and the modification of proteins hardly influenced or damaged the particle size and the morphology of PS nanoparticles. The insets of the TEM images show the PS nanoparticles, AIE_490_NP, and AIE_490_NP‐BSA under white light and UV irradiation, highlighting the strong fluorescence of AIE_490_NP and AIE_490_NP‐BSA and indicating the successful encapsulation of AIE_490_. The PS nanoparticles AIE_490_NP and AIE_490_NP‐BSA were elementally analyzed using X‐ray photoelectron spectroscopy (XPS) (Figure S12, Supporting Information). The higher nitrogen (Figure S13b, Supporting Information) content in AIE_490_NP than in the empty PS particles also proved the encapsulation of AIE_490_. The significant increases in the oxygen (Figure S13a, Supporting Information), nitrogen (Figure S13b, Supporting Information), and sulfur (Figure S13d, Supporting Information) contents demonstrated the successful conjugation of BSA on the surface of AIE_490_NP.

### 2.2 Preparation and Characterization of AuNP‐ACE2

**Figure 2:**
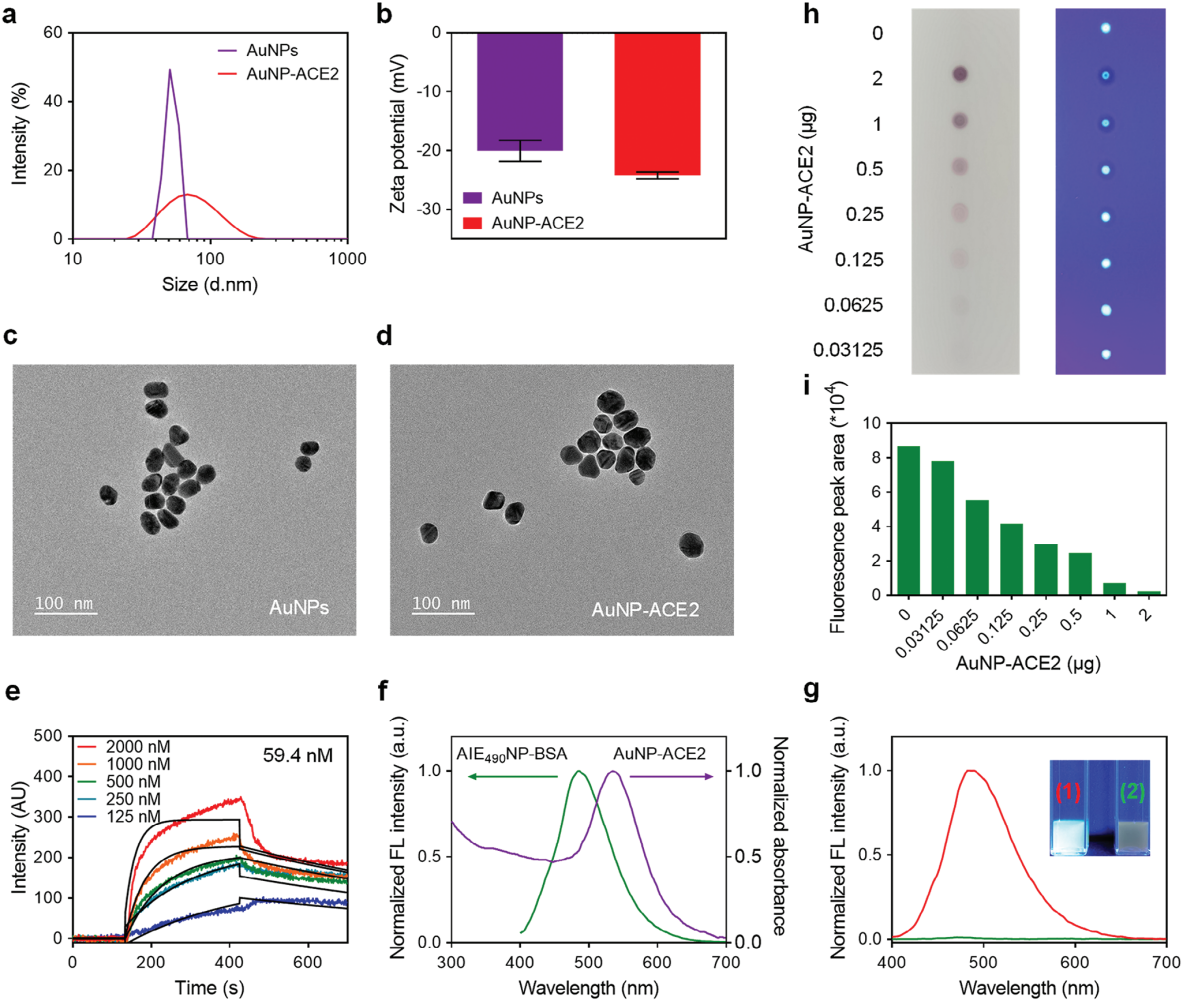
The hydrodynamic diameter a) and zeta potential b) of AuNP and AuNP‐ACE2. The TEM images of AuNP c) and AuNP‐ACE2 d). e) The affinity constant of AuNP‐ACE2 toward immobilized RBD on 3D dextran chip. f) The fluorescence spectra of AIE_490_NP‐BSA and absorption of AuNP‐ACE2. g) The fluorescence intensity of AIE_490_NP‐BSA (100 µg mL^−1^ diluted in water) mixed with 0 µg (1) or 200 µg (2) AuNP‐ACE2. h) The spots of the AIE_490_NP‐BSA/AuNP‐ACE2 mixtures with different amounts of AuNP‐ACE2 on the NC membrane under white light (left) and UV light (right, *λ*_ex_ = 365 nm). (i) The fluorescence signals of the spot brightness in (h).

AuNP‐ACE2 conjugates were prepared through electrostatic attraction between the negatively charged AuNPs and the positively charged ACE2 protein in a weakly basic environment. The differences in hydrodynamic diameter (**Figure** **2**a) and zeta potential (Figure 2b) between AuNP (50.7 nm and−20.1 mV) and AuNP‐ACE2 (68.1 nm and−24.2 mV) indicated the successful combination of AuNP and ACE2 protein. The TEM images of the AuNPs (Figure 2c) and AuNP‐ACE2 (Figure 2d) suggested that the conjugation of ACE2 did not damage the colloidal structural stability of AuNPs. The affinity between AuNP‐ACE2 and RBD was determined at 59.4 nm (Figure 2e) using surface plasmon resonance (SPR). As shown in Figure 2f, AuNP‐ACE2 exhibited broad absorption in the wavelength range and maximum absorption at 535 nm, consistent with unlabeled AuNPs (Figure S14, Supporting Information). The maximum fluorescence of AIE_490_NP‐BSA was observed at 490 nm, partially coinciding with the absorption of AuNP‐ACE2, suggesting a strong possibility of a FRET quenching effect between them. The fluorescence intensity of AIE_490_NP‐BSA mixed with different amounts of AuNP‐ACE2 further verified the FRET effect (Figure S15, Supporting Information). As the amount of AuNP‐ACE2 increased from 0 to 200 µg, the fluorescence intensity of AIE_490_NP‐BSA gradually weakened until it disappeared (Figure 2g). To ensure the FRET quenching effect could be performed on the NC membrane, 10 µL of the AIE_490_NP‐BSA/AuNP‐ACE2 mixtures with different amounts of AuNP‐ACE2 were sequentially dropped onto the NC membrane. Then each spot brightness on the NC membrane was measured using the test strip reader to obtain the fluorescence signal. The spot brightness of AIE_490_NP‐BSA also decreased with increasing amounts of AuNP‐ACE2 (Figure 2h,i), indicating the feasibility of weakening the fluorescence of AIE_490_NP‐BSA by AuNP‐ACE2 on the NC membrane.

### 2.3 The Mechanism of the FQ‐LFIA

**Figure 3:**
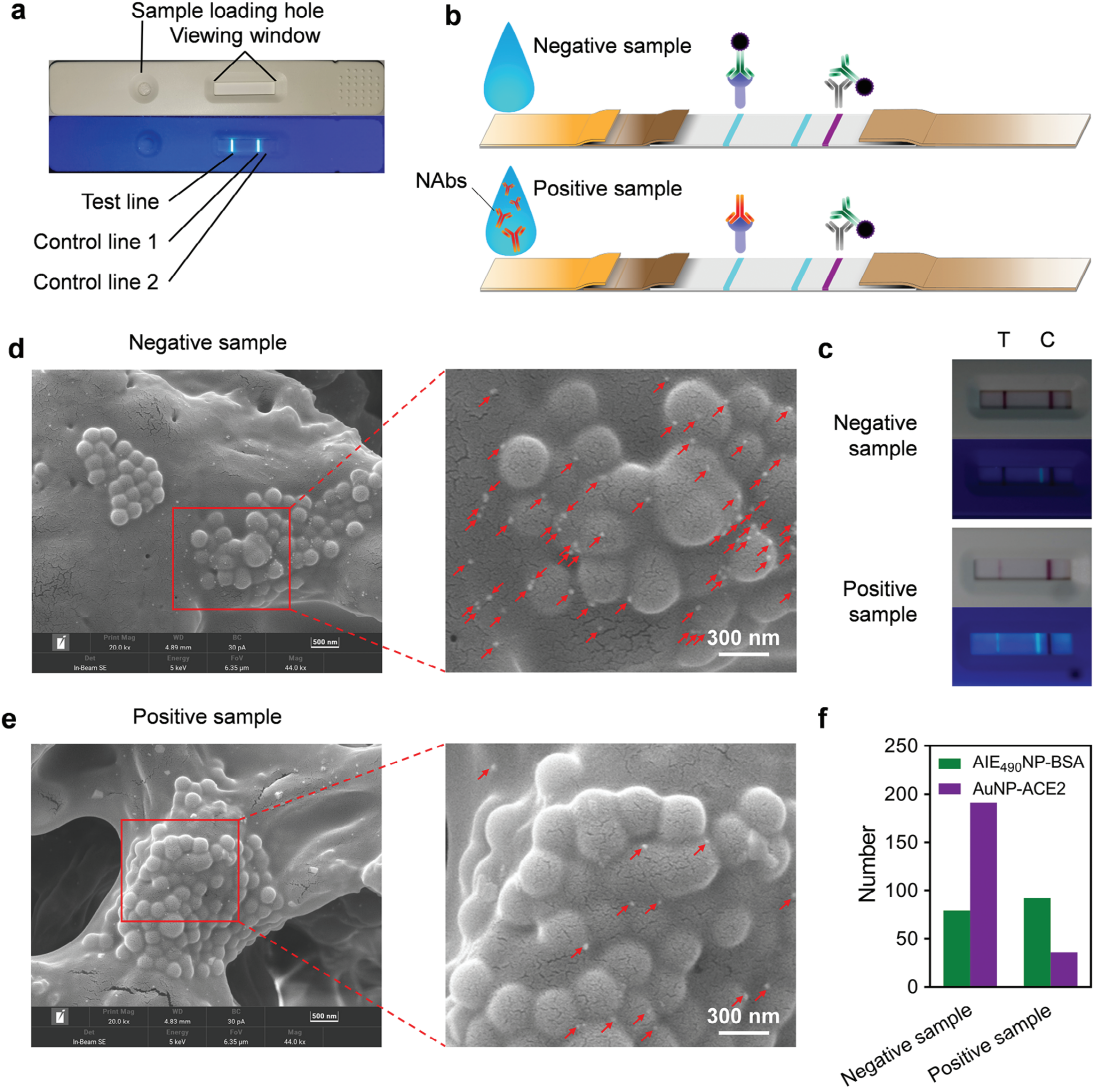
a) The product pictures of the established FQ‐LFIA under white light (upper) or UV light (down, *λ*_ex_ = 365 nm). b) The working mechanism of the FQ‐LFIA. c) The pictures of the visual detection results for negative or positive samples under white light (upper) or UV light (down, *λ*_ex_ = 365 nm). The SEM images of the T line on the FQ‐LFIA test strip for detecting negative d) or positive samples e); the small particles pointed by the red arrow are AuNPs. f) The total counts of the AIE_490_NP‐BSA and AuNP‐ACE2 in (d) and (e).

The established FQ‐LFIA was performed as a competitive lateral flow immunoassay based on competitive binding between ACE2 and anti‐SARS‐CoV‐2 NAbs to the RBD protein. **Figure** **3**a shows the product of the FQ‐LFIA under white light (upper) or UV light (down, *λ*_ex_ = 365 nm). The green fluorescence of the T line and control line 1 originated from the AIE_490_NP coated on them. As shown in Scheme S1 (Supporting Information), the RBD/AIE_490_NP‐BSA mixture was coated on the NC membrane as the T line, while the separate AIE_490_NP‐BSA was coated at a distance of 10 mm from the T line as control line 1 for fluorescence detection. Meanwhile, for visual observation, anti‐ACE2 IgG was coated 3 mm backward as control line 2. Scanning electron microscope (SEM) images of the uncoated NC membrane (Figure S16a, Supporting Information) and the coated test (Figure S16b, Supporting Information) or control line (Figure S16c, Supporting Information) positions show clear nanoparticle structures after coating with AIE_490_NP‐BSA. As shown in Figure 3b, when using the FQ‐LFIA strip, 100 µL of sample buffer containing anti‐SARS‐CoV‐2 NAbs was added dropwise to the sample loading hole and migrated to the conjugated pad under capillary action. The buffer then carried the AuNP‐ACE2 fixed on the pad and continued to flow toward the NC membrane. AuNP‐ACE2 was then recognized and bound by the RBD once it reached the T line, forming a purple‐red band at the T line and quenching the fluorescence initially present at the T line under the FRET effect. Meanwhile, the anti‐SARS‐CoV‐2 NAbs in the buffer recognized and blocked the RBD, reducing the aggregation of AuNP‐ACE2 at the T line and resulting in lower fluorescence quenching. The SEM image of the T line on the strips used to detect a positive sample (Figure 3f) showed the presence of more AuNP‐ACE2 on the T line (Figure 3d) than that used to detect a negative sample (Figure 3e). Hence, a higher anti‐SARS‐CoV‐2 NAbs concentration led to a weaker purple‐red band under white light and brighter fluorescence under UV light at the T line (Figure 3c). Subsequently, the AuNP‐ACE2 not bound to the T line migrated further to control line 2 and was captured by the anti‐ACE antibodies coated on it to form a purple‐red band on control line 2. Fluorescent control line 1 was also introduced. Throughout the test, the fluorescence signal intensity of control line 1 remained relatively stable. After the detection process, the strip was measured using a portable fluorescence reader to obtain the fluorescence signals of the T line (H_T_) and control line 1 (H_C1_). The ratio of H_T_–H_C1_ was further used as the final result to fit the correlation curve.

### 2.4 Optimization of the Detection Conditions

**Figure 4:**
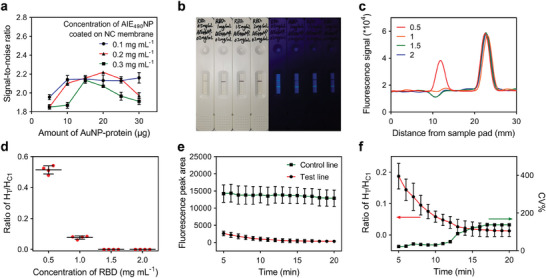
a) The signal‐to‐noise ratio of the detection results to optimize the concentration of AIE_490_NP‐BSA and amount of AuNP‐ACE2; each point was obtained with three replicates. b) The picture of visual detection results in a measured sample buffer using strips coated with different concentrations (0.5, 1, 1.5, and 2 mg mL^−1^) of RBD protein on the T line under white light (left) and UV light (right, *λ*_ex_ = 365 nm). c) The readout curve of strips (shown in (b)) using a portable fluorescence reader. d) The H_T_/H_C1_ ratio of detection results from using strips coated with different concentrations of RBD protein on the T line. e) The trends of H_T_ and H_C1_ with increasing incubation time from 5 to 20 min since sample buffer loading; each point was obtained with five replicates. f) The trends of H_T_/H_C1_ ratio and CV % with increasing incubation time from 5 to 20 min since sample buffer loading; each point was obtained with five replicates.

The performance of FQ‐LFIA highly depends on the detection conditions, including the concentration of AIE_490_NP‐BSA or RBD protein coated on the T line, the amount of AuNP‐ACE2 immobilized on the conjugate pad, and the incubation time from loading sample to measuring strip. As the concentration of AIE_490_NP‐BSA coated on the detection line and the amount of AuNP‐ACE2 on the binding pad dynamically affected the fluorescence intensity of the detection line after the test, these two detection conditions were optimized simultaneously. As shown in Figure S17a (Supporting Information), the concentration of AIE_490_NP‐BSA was optimized from 0.1, 0.2, and 0.3 mg mL^−1^. As the concentration of the fluorescent nanoparticles coated on the NC membrane increased, the fluorescence signal of the T line also increased (Figure S17b, Supporting Information), indicating that H_T_ was positively correlated with the amount of coated AIE_490_NP‐BSA. Meanwhile, the conjugate pads fixed with different amounts (5–30 µg) of AuNP‐ACE2 were used with the previously mentioned NC membrane coated with different concentrations of AIE_490_NP‐BSA on the T line to assemble the FQ‐LFIA strips. To eliminate the differences in the degree of fluorescence quenching caused by the increasing amount of AuNP‐ACE2, test strips with the same amount of AuNP‐BSA fixed on the conjugate pad were used as controls. With an increase in AuNP‐ACE2/BSA usage, the fluorescence of the T line in the control group slowly decreased because of the residue of AuNPs on the NC membrane, whereas that of the experimental group decreased significantly (Figure S18, Supporting Information). The signal‐to‐noise ratio (**Figure** **4**a) was calculated to evaluate the performance of each experimental group using the following equation:

(1) $$\mathrm{Signal}-\mathrm{to}-\mathrm{noise}\mathrm{ratio}=\frac{H_{T0}\;-\;H_{T1}}{H_{T0\;}-\;H_{T2}}$$

where H_T0_ is the fluorescence peak area of the T line before detection, and H_T1_ and H_T2_ are the areas of the T line in the experimental and control groups, respectively. A higher signal‐to‐noise ratio indicates that the residual AuNPs have a lesser effect on the results. The concentration of AIE_490_NP‐BSA coated on the T line and the amount of AuNPs immobilized on the conjugate pad were ultimately optimized as 0.2 mg mL^−1^ and 20 µg, respectively. Control line 1, coated with the same concentration of AIE_490_NP‐BSA, was introduced to counteract the influence of random factors on detection results.

The concentration of RBD protein coated on the T line was optimized at 0.5, 1, 1.5, and 2 mg mL^−1^, referencing the fluorescence signal of the T line and the H_T_/H_C1_ ratio. The test strips with different concentrations of RBD (0.5–2 mg mL^−1^ from left to right) coated on the T line were used to measure the sample buffer and the visual results under white light (left) or UV light (right, *λ*_ex_ = 365 nm) (Figure 4b; Figure S19, Supporting Information). The strips were then analyzed using a fluorescence reader to obtain the fluorescence readout curve (Figure 4c; Figure S19 and , Supporting Information), H_T_ (Figure S20a, Supporting Information), H_C1_ (Figure S20b, Supporting Information), and the H_T_/H_C1_ ratio (Figure 4d). The visual results and readout curve showed that the fluorescence signal of the T line continued to decrease as the RBD protein concentration increased from 0.5 to 1.5 mg mL^−1^ and remained constant as the concentration further increased to 2 mg mL^−1^. The concentration of RBD coated on the T line was better to be higher to ensure the detection range and sensitivity requirements of FQ‐LFIA. However, since the reader cannot accurately calculate the fluorescence peak signal below 0 when the coated RBD concentration is 1.5 or 2 mg mL^−1^, the large amount of AuNPs enriched on the T line causes the fluorescence signal to be lower than 0 and miscalculated to be 0 by the reader, causing the fluorescence signal of the T line to fail to correlate with the amount of AuNPs.

The incubation time of the FQ‐LFIA was optimized by measuring sample buffer using the strips with RBD protein (1 mg mL^−1^) and AIE_490_NP (0.2 mg mL^−1^) on the T line, single AIE_490_NP (0.2 mg mL^−1^) on control line 1, and AuNP‐ACE2 (20 µg) immobilized on the conjugate pad. As shown in Figure 4e, the fluorescence peak area of the T line (red) reached a low level at 5 min after loading the buffer, owing to the good binding ability of AuNP‐ACE2 to the RBD and the ability of AuNPs to quench the fluorescence of AIE_490_NP, and further decreased gradually with an increase in reaction time to 20 min, while that of control line 1 (green) slowly decreased under the influence of free AuNPs on the NC membrane. The calculated H_T_/H_C1_ ratio exhibited a rapid downward trend over the 5–13 min range and reached a plateau at 13 min (Figure 4f, red curve). 10 min was selected as the incubation time between sample loading and fluorescence signal measurement to ensure the robustness of the assay based on the SD (Figure 4f, green curve) of the H_T_/H_C1_ ratio at each time point.

### 2.5 Dose‐Response Curve and Detection Performance of the FQ‐LFIA

**Figure 5:**
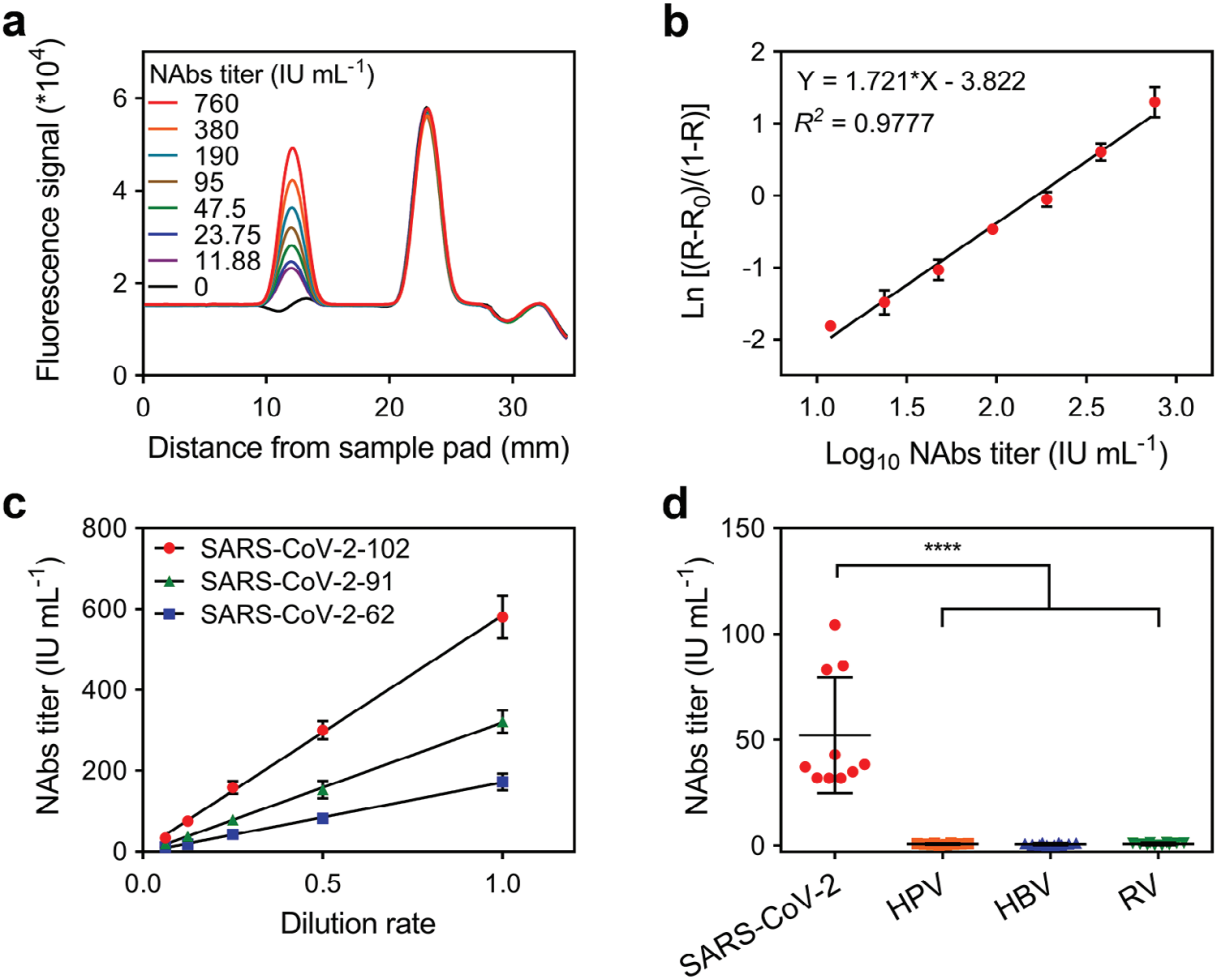
a) The readout curve of the test strips used for detecting the calibrators. b) The dose‐response curve of the FQ‐LFIA; each point was obtained with three replicates. c) The dilution test results of the FQ‐LFIA; each point was obtained with three replicates. d) The detection results of the serum samples of individuals vaccinated with SARS‐CoV‐2 (n = 10), HPV (n = 10), HBV (n = 10), and RV (n = 10).

The dose‐response curve of the FQ‐LFIA was obtained by detecting the calibrators and calculating the H_T_/H_C1_ ratio. The calibrators were prepared by continuously diluting a positive serum sample with anti‐SARS‐CoV‐2 NAbs titer of 760 IU mL^−1^ in hormone‐free human serum to different titers of 760, 380, 190, 95, 47.5, 23.75, and 11.88 IU mL^−1^. The calibrators were then diluted using the sample buffer and loaded onto the test strips to obtain a fluorescence readout curve (**Figure** **5**a; Figure S21, Supporting Information), which included the H_T_, H_C1_, and H_T_/H_C1_ ratios. The dose‐response curve was constructed to be logit (Y) = 1.721 * log (X)–3.822 with correlation coefficient (*R* ^2^) = 0.9777 by plotting logit (Y) against the logarithm of the NAbs titer (X). Logit (Y) was calculated as ln [(R−R_0_)/(1−R)], where R is the H_T_/H_C1_ ratio of the calibrator and R_0_ is that of the sample buffer (Figure 5b). At the same time, to evaluate the possibility of FQ‐LFIA detection results under visual observation, image‐j was used to analyze the grayscale of the purple‐red band formed by AuNPs aggregation at the T line on the strips under white light, which was significantly correlated with the anti‐SARS‐CoV‐2 NAbs titer (Figure S22, Supporting Information) and performed an LoD of 48.72 IU mL^−1^, indicating the feasibility of visual interpretation of FQ‐LFIA results by the naked eye.

The performance of the FQ‐LFIA was evaluated based on reproducibility, recovery, and specificity. Three serum samples with high (SARS‐CoV‐2‐102, 615 IU mL^−1^), medium (SARS‐CoV‐2‐91, 307 IU mL^−1^), and low (SARS‐CoV‐2‐62, 163 IU mL^−1^) NAbs titers were selected as quality control samples (QCs). Reproducibility was evaluated by detecting QCs with 10 replicates within a day as an intra‐assay and five replicates each day for five continuous days as an inter‐assay. The intra‐ and inter‐assay coefficients of variation (CV %) were 6.76–9.61% and 9.48–12.17% (**Table** **1**), respectively; all below 15%, suggesting an acceptable reproducibility of FQ‐LFIA for practical application. In addition, QCs with continuous dilution were detected to assess the recovery of FQ‐LFIA. The dilution test showed that the detection results of each continuously diluted QC performed good linearity (Figure 5c), indicating an excellent recovery of FQ‐LFIA. Serum samples from 10 individuals vaccinated with human papillomavirus (HPV), 10 with hepatitis B virus (HBV), and 10 with rabies virus (RV) were tested using the FQ‐LFIA strips and compared with 10 serum samples from individuals vaccinated with SARS‐CoV‐2 to evaluate the specificity of the FQ‐LFIA. As shown in Figure 5d, The FQ‐LFIA showed excellent specificity for detecting anti‐SARS‐CoV‐2 NAbs in vaccinated serum samples.

**Table 1:** The intra‐ and inter‐assay of the FQ‐LFIA.

	Sample NO.	Titer (IU mL^−1^)	Mean ± SD (IU mL^−1^)	CV (%)
Intra‐assay (n = 10)	SARS‐CoV‐2‐102	615	598.98 ± 40.48	6.76
	SARS‐CoV‐2‐91	307	305.21 ± 27.96	9.16
	SARS‐CoV‐2‐62	163	167.10 ± 16.06	9.61
Inter‐assay (n = 5)	SARS‐CoV‐2‐102	615	595.64 ± 56.48	9.48
	SARS‐CoV‐2‐91	307	302.63 ± 36.82	12.17
	SARS‐CoV‐2‐62	163	161.21 ± 16.56	10.27

### 2.6 Practical Application of the FQ‐LFIA

**Figure 6:**
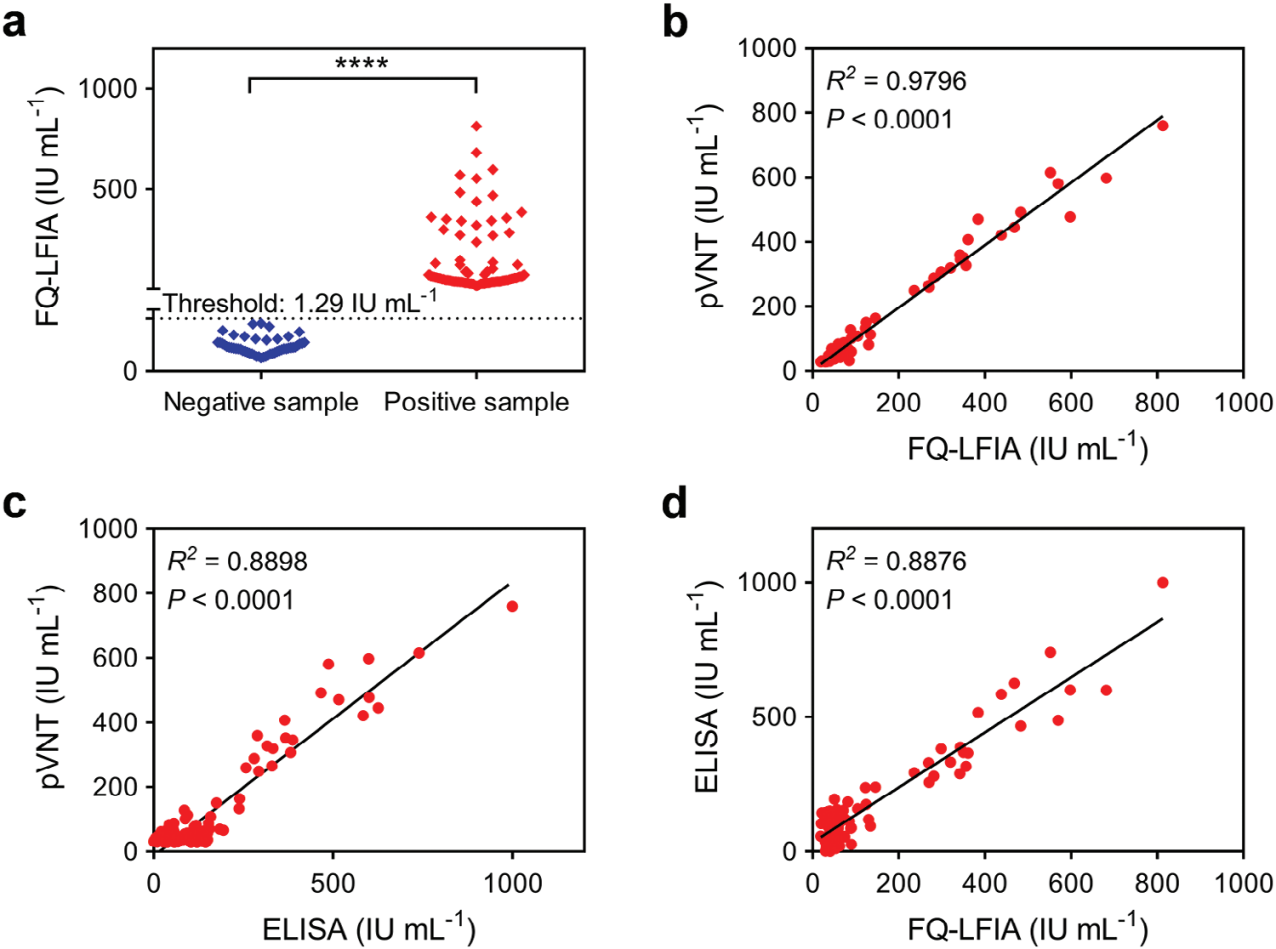
a) Results of the 103 positive and 50 negative samples detected using the FQ‐LFIA (the dotted line represents the LoD). b) Comparison between the FQ‐LFIA and pVNT in detecting the 103 positive serum samples. c) Comparison between commercial ELISA kit and pVNT. d) Comparison between FQ‐LFIA and commercial ELISA kit.

A total of 50 negative samples collected before COVID‐19 infection and 103 positive samples from individuals vaccinated with SARS‐CoV‐2 were tested using the FQ‐LFIA strips and compared with the pVNT and commercial ELISA (Figure S23, Supporting Information) results to evaluate the performance of FQ‐LFIA in practical applications. The FQ‐LFIA effectively identified positive and negative samples (*P* < 0.0001, **Figure** **6**a). Meanwhile, by defined as mean plus 3*SD of the 50 negative NAbs titer results, the LoD of the FQ‐LFIA was 1.29 IU mL^−1^. The anti‐SARS‐CoV‐2 NAbs titers of the serum samples were compared among the three methods (Table S5, Supporting Information). The FQ‐LFIA results were significantly correlated with the pVNT (*R* ^2^ = 0.9796, *P* < 0.0001) and commercial ELISA kit (*R* ^2^ = 0.8876, *P* < 0.0001); commercial ELISA kit results were also statistically correlated (*R* ^2^ = 0.8898, *P* < 0.0001) with the pVNT results, suggesting that FQ‐LFIA has a better correlation with pVNT than with commercial ELISA. The calibrators were tested using AIE_490_NP‐based LFIA strips to compare the detection performance of the FQ‐LFIA and the AIE_490_NP‐based LFIA established in our previous work (Scheme S2, Supporting Information). Under optimized reaction conditions (Figure S24c,d, Supporting Information), the AIE_490_NP‐based LFIA was effective in quantitatively detecting anti‐SARS‐CoV‐2 NAbs (*R* ^2^ = 0.9577) (Figure S24f, Supporting Information) with an LoD of 11.06 IU mL^−1^ (Figure S24g, Supporting Information). In comparison, the FQ‐LFIA performed better than the AIE_490_NP‐based LFIA in the quantitative detection of anti‐SARS‐CoV‐2 NAbs, indicating that AIE_490_NP could perform better in a “turn‐on” FQ‐LFIA assisted by AuNP. The efficacy of FQ‐LFIA for the quantitative detection of anti‐SARS‐CoV‐2 variants NAbs titers was evaluated considering the continuing evolution of SARS‐CoV‐2 and the emergence of new circulating variants. A total of 56 serum samples were analyzed by pVNT for anti‐SARS‐CoV‐2 Delta‐variant NAbs titers (Table S6, Supporting Information). Although the anti‐SARS‐CoV‐2 Delta‐variant NAbs titers detected by pVNT are lower than those of the anti‐SARS‐CoV‐2 wild‐type strain detected by FQ‐LFIA (Figure S25a, Supporting Information), their results exhibit a strong correlation (*R* ^2^ = 0.8584, *P* < 0.0001, Figure S25b, Supporting Information), indicating that the detection results obtained by FQ‐LFIA could evaluate anti‐SARS‐CoV‐2 Delta‐variant NAbs titers as a reference.

## 3 Discussion

In this study, by combining AuNP and AIE_490_NP, we established an FQ‐LFIA with a “turn‐on” mode to detect the anti‐SARS‐CoV‐2 NAbs titer in human serum using both visual assessment and a portable fluorescence strips reader. Unlike our previously reported LFIA for the qualitative analysis of NAbs titers,^[^ ^14^ ^]^ we achieved the quantitative detection of NAbs titers using the established FQ‐LFIA by selecting a serum sample with high antibody titers as the standard. As an immunoassay method that does not require live viruses or cell culture,^[^ ^24^ ^]^ LFIA has made extensive progress in detecting the SARS‐CoV‐2 antigen or anti‐SARS‐CoV‐2 total antibodies because of its simplicity, low instrument price, and no complex operation.^[^ ^25^, ^26^ ^]^ However, LFIA methods that can quantitatively detect anti‐SARS‐CoV‐2 NAbs have rarely been reported. Distinct from the detection of anti‐SARS‐CoV‐2 total antibodies using indirect LFIA with SARS‐CoV‐2 protein fragment coating as the T line and labeled anti‐human IgG as the reporter,^[^ ^27^ ^]^ the LFIA detecting anti‐SARS‐CoV‐2 NAbs is commonly established as a competitive method, performed through the competitive combination of ACE2 and NAbs for the RBD fragment. High titers of neutralizing antibodies in serum are more resistant to viral attacks.^[^ ^28^ ^]^ This study combined AuNP and AIE_490_NP applications in FQ‐LFIA, enabling rough observation of the NAbs titer by visually observing the AuNP purple‐red band under white light. In the presence of the purple‐red AuNP band on the control line two, the lighter the color of the purple‐red AuNP band of the detection line on the test strip, the higher the neutralizing antibody titer in the serum sample. Subsequently, a portable fluorescence reader was used to obtain the fluorescence signal of the T line and control line 1 under a UV lamp to construct the dose‐response curve between the fluorescence signal and the NAbs titers for the quantitative detection of anti‐SARS‐CoV‐2 NAbs.

As a method of combining naked‐eye visual results and fluorescence reading results, FQ‐LFIA can help vaccinated people and patients infected with SARS‐CoV‐2 self‐monitor the titer of anti‐SARS‐CoV‐2 NAbs in the body without instruments, which plays a crucial role in the current normalization of COVID‐19.^[^ ^29^ ^]^ The protection of anti‐SARS‐CoV‐2 NAbs wanes over 6–9 months after two vaccine doses,^[^ ^30^ ^]^ and the NAbs attenuate faster in older adults, children, and individuals who are immunocompromised,^[^ ^31^ ^]^ which means that the NAbs titers vary significantly between individuals over some time after vaccination. Booster vaccination is an effective way to attenuate NAbs and reorganize immune resistance.^[^ ^32^ ^]^ However, owing to differences in individual immunity, some vaccinated individuals lose the protection of NAbs before booster vaccination, and this window period may be long. Hence, an accurate, rapid, and easy‐to‐operate method for detecting anti‐SARS‐CoV‐2 NAbs is the key to guiding individuals to schedule booster doses and maintain effective resistance against SARS‐CoV‐2.

The established FQ‐LFIA provides a new strategy for individuals vaccinated with SARS‐CoV‐2 or those who are infected to self‐evaluate anti‐SARS‐CoV‐2 NAbs titers and immune resistance against SARS‐CoV‐2. In addition, FQ‐LFIA has also exhibited the ability to roughly assess the anti‐SARS‐CoV‐2 Delta‐variant strain NAbs titers. We believe that FQ‐LFIA could perform better in the detection of NAbs titers toward various SARS‐CoV‐2 variants by replacing the RBD recombinant protein of wild‐type strain used in this study with that of variant strains in follow‐up research. Although the established method can enable users to judge NAbs titers by visual observation, the failure to provide accurate quantitative detection of NAbs titers without detection equipment hinders the actual implementation of this method for point‐of‐care testing (POCT). In follow‐up research, we expect to achieve this goal through the use of smartphones or image sensors and further promote the generalization of such methods in POCT and home self‐monitoring. With further research on such methods, we plan to establish more FQ‐LFIAs for dual‐mode detection, naked‐eye observation, and fluorescence reading of disease markers such as tumor indicators, inflammatory indicators, and food residue toxins, which also require immediate rapid detection. With the application of different fluorescent AIEgen‐chelated nanoparticles, the detection of multiple related disease markers using the same strip is possible.

## 4 Conclusion

In conclusion, we have successfully established a “turn‐on” FQ‐LFIA to quantitatively detect anti‐SARS‐CoV‐2 NAbs in human serum within 10 min. The dose‐response curve of the established FQ‐LFIA was calculated as logit (Y) = 1.721 * log (X)‐1.973 (*R* ^2^ = 0.9777). To evaluate the performance of the established FQ‐LFIA, 103 positive and 50 negative serum samples were analyzed using FQ‐LFIA and compared with the pVNT and commercial ELISA kit. The FQ‐LFIA exhibited better correlation (*R* ^2^ = 0.9796, *P* < 0.0001) and LoD (1.29 IU mL^−1^) than the commercial ELISA kit (*R* ^2^ = 0.8898, *P* < 0.0001) with the standard pVNT. FQ‐LFIA exhibited better detection performance than AIE_490_NP‐based LFIA, which uses only AIE_490_NP as the fluorescence label. The intra‐(6.76–9.61%) and inter‐assay (9.48–12.17%) of the established FQ‐LFIA suggest an acceptable reproducibility. Thus, the established FQ‐LFIA has strong potential as an alternative method for quantitatively and rapidly detecting anti‐SARS‐CoV‐2 neutralizing antibodies. Whether the FQ‐LFIA can be applied to other diagnoses deserves further research.

## 5 Experimental Section

### Clinical Serum Samples

Serum samples were collected from Southern Medical University Nanfang Hospital, including 103 positive samples obtained from participants who were vaccinated with the anti‐SARS‐CoV‐2 vaccine and 50 negative samples obtained before COVID‐19 infection. All serum samples were pre‐measured to obtain anti‐SARS‐CoV‐2 NAbs titers using the pVNT method. The titers and background information of these samples were shown in Tables S1 and S2 (Supporting Information). This study was approved by the Southern Medical University Nanfang Hospital (Guangzhou, China) (ethics approval number: NFEC‐2023‐148). All human serum experiments were performed according to The Code of Ethics of the World Medical Association (Declaration of Helsinki).

### Synthesis and Characterization of AIE_490_NP

The AIE_490_NP was prepared as described in the previous study.^[^ ^14^ ^]^ The hydrodynamic diameter of the carboxyl‐modified PS nanoparticles used in this study was 300 nm. Multiple swelling procedures were performed to encapsulate more AIEgens in PS nanoparticles to obtain a brighter fluorescence of AIE_490_NP.

The spectral properties of AIE_490_ and AIE_490_NP were measured using a UV‐vis spectrophotometer (Shimadzu, Japan) and a Lumina spectrophotometer (Thermo Fisher Scientific Inc., USA), respectively. The preparation of AIE_490_NP was evidenced by the zeta potential, hydrodynamic diameter (Nano‐ZS90 ZetaSizer, Malvern Panalytical Ltd., UK), and TEM images (H‐7500 transmission electron microscope, Hitachi Co., Ltd., Japan). The PLQYs were measured using quinine sulfate (PLQY = 55% in 0.1 M H_2_SO_4_) as a reference. The optical stability of AIE_490_ and AIE_490_NP was evaluated by monitoring the fluorescence intensity of samples exposed to white light or stored at different temperatures and pH levels for a week. The morphological stability of AIE_490_NP was assessed through changes in the hydrodynamic diameter.

### Preparation of AuNP‐ACE2 and Modified AIE_490_NP

AuNPs and ACE2 were combined via electrostatic adsorption. Initially, 1 mg of AuNP was resuspended in 500 µL of K_2_CO_3_ solution (0.1 m, pH 9.0) to adjust the pH to 9.0. ACE2 (100 µg) protein was condensed using an Ultracel‐50 kDa membrane centrifuge filter unit and diluted in 500 µL of K_2_CO_3_ solution (0.1 m, pH 9.0). The ACE2 protein was then added dropwise to the treated AuNPs for 5 min, followed by 10 mg of BSA; and incubation with oscillation was continued for 10 min. Subsequently, the mixture was centrifuged to remove unlabeled ACE2. The AuNP‐ACE2 conjugate was resuspended in 100 µL of labeled antibody storage buffer.

The surface of AIE_490_NP was modified with a carrier protein to ensure a stable coating on the NC membrane. BSA was selected as the carrier protein for combination with AIE_490_NP using the EDC/NHS method. Initially, 1 mg of AIE_490_NP was resuspended in 400 µL of activating buffer, followed by adding 5 µmol of NHS and 5 µmol of EDC. After 30 min of vortex oscillation at room temperature, the activated AIE_490_NP was centrifuged to remove the activating buffer containing the remaining unreacted EDC/NHS and washed twice with washing buffer. The precipitated AIE_490_NP was resuspended in 1 mL of binding buffer containing 10 mg of BSA. The mixture was vortex oscillated at 4 °C overnight for covalent coupling of BSA and NHS‐ester on the surface of activated AIE_490_NP. After oscillation, AIE_490_NP‐BSA was centrifuged to remove the binding buffer containing uncoupled BSA and washed twice with washing buffer. AIE_490_NP‐BSA was suspended in 100 µL of labeled antibody storage buffer.

The AuNP‐ACE2 and AIE_490_NP‐BSA were stored at 4 °C till further use.

### Pretreatment of the Sample, Conjugate, and Absorbent Pads

The sample and conjugate pads were 300 × 21 mm and 300 × 12 mm pieces, respectively, which were cut from fiberglass paper followed by soaking in the equivalent treatment buffer for 2 h at room temperature and drying at 37 °C for 24 h using a vacuum shelf dryer. The absorbent pad was 300 × 26 mm pieces cut from the absorbent paper with no additional treatment. All pads were stored in a drying cabinet until further use.

### Preparation of FQ‐LFIA

The FQ‐LFIA test strip was composed of a backing plate, NC membrane, conjugate pad, sample pad, and absorbent pad. The recombinant RBD protein was mixed with AIE_490_NP‐BSA diluted in the coating buffer. The anti‐ACE2 antibody was diluted in the coating buffer for coating use. The RBD/AIE_490_NP‐BSA mixture, prediluted AIE_490_NP‐BSA, and anti‐ACE2 IgG were sprayed uniformly onto the NC membrane as the T line, control line 1, and control line 2, respectively. AuNP‐ACE2 was diluted with the labeled antibody dilution buffer and sprayed onto the conjugate pad. The treated NC membrane and conjugate pad were then fixed on an adhesive backing plate and dried in a vacuum oven at 37 °C for 12 h. The sample and absorbent pads were then sequentially pasted onto the backing plate to ensure that the sample buffer flowed from the sample pad to the absorbent pad. The composition was then cut into several 4 mm wide pieces to obtain the final FQ‐LFIA strips. Each strip was fitted into a shell with a sample‐loading hole and a viewing window. The assembled strips were then stored in a drying cabinet until further use.

### Pseudovirus‐Based Virus Neutralization Test

The anti‐SARS‐CoV‐2 NAbs standard, negative control, and serum samples were diluted in 96‐well plates with complete DMEM to reach a volume of 75 µL per well, and then 25 µL of pseudovirus suspension was added to each well. The plates were gently shaken to mix well and then incubated in a 5% CO_2_ incubator at 37 °C for 60 min. After incubation, the precultured human ACE2 overexpression stable HEK293 cells were digested and resuspended in complete DMEM and added to these 96‐well plates with 100 µL of the cell suspension per well. The plates were shaken gently to mix well and incubated in a 5% CO_2_ incubator at 37 °C for 48 h. The 96‐well plates were removed and the medium was discarded followed by adding 100 µL of detection reagent per well. After mixing, the plate was incubated for 2 min at room temperature. The luminescence values (RLU) of the wells were obtained using a luminescence meter.

### Enzyme‐Linked Immunosorbent Assay

The ELISA method references the protocol in the ELISA kit. The ELISA microplate was pre‐coated with anti‐rabbit IgG. The calibrators were obtained by serially diluting the standard serum sample using the dilution buffer to the titers of 760, 380, 190, 95, 47.5, 23.75, 11.88 IU mL^−1,^ and the rabbit Fc‐tagged SARS‐CoV‐2 RBD and horse radish peroxidase (HRP) labeled ACE2 were reconstituted with 6 mL of dilution buffer. When testing using the ELISA kit, the microplate was initially washed five times using the washing buffer, then 50 µL of serum sample/calibrator and 50 µL of rabbit Fc‐tagged SARS‐CoV‐2 RBD reagent were added to the microplate in succession. After incubation for 60 min at room temperature, 50 µL of HRP‐ACE2 reagent was added to the microplates followed by another incubation for 30 min at room temperature. Subsequently, the microplates were emptied and washed five times using the washing buffer. Then each microplate was added with 175 µL of 3,3′,5,5′‐tetramethylbenzidine (TMB) substrate solution and incubated for 30 min at room temperature. Finally, the microplates were added with 75 µL of stop solution for each hole and measured using a Multiskan SkyHigh plate reader to obtain the optical density at 450 nm (OD_450_).

### Statistical Analysis

Statistical data were analyzed using GraphPad Prism 9.5.1. All bar and X‐Y plots were presented as mean ± SD obtained by repeated measures. Statistical analysis was performed using a one‐way repeated measures analysis of variance (ANOVA) to compare the results of different sample groups. Differences between positive and negative groups were considered statistically significant with a *P* value < 0.0001. The sample sizes of each experiment were indicated in the figure legends.

## Conflict of Interest

The authors declare no conflict of interest.

## Supporting information

Supporting Information

## Data Availability

Research data are not shared.
