# Supplementary material for: Fluorescence‐Quenching Lateral Flow Immunoassay for “Turn‐On” and Sensitive Detection of Anti‐SARS‐Cov‐2 Neutralizing Antibodies in Human Serum
Source: Adv Sci (Weinh). 2023 Nov 30;11(4):2305774. doi: 10.1002/advs.202305774 (PMC10811470; doi:10.1002/advs.202305774)
Supplement: Supplementary file 1 — Supporting Information [file ADVS-11-2305774-s001.pdf]

ADVANCED SCIENCE
Open Access

Supporting Information

for *Adv. Sci.*, DOI 10.1002/advs.202305774

Fluorescence-Quenching Lateral Flow Immunoassay for "Turn-On" and Sensitive Detection of Anti-SARS-Cov-2 Neutralizing Antibodies in Human Serum

Lun Bian, Qiangqiang Fu, Zhuoheng Gan, Ze Wu, Yuchen Song, Yufeng Xiong, Fang Hu* and Lei Zheng**

Fluorescence-quenching lateral flow immunoassay for "turn-on" and sensitive detection of anti-SARS-CoV-2 neutralizing antibodies in human serum

Lun Bian, Qiangqiang Fu, Zhuoheng Gan, Ze Wu, Yuchen Song, Yufeng Xiong, Fang Hu*, and Lei Zheng**

L. Bian, Z. Gan, Y. Song, F. Hu
Biomaterials Research Center
School of Biomedical Engineering
Southern Medical University
510515 Guangzhou, China
Email: hufang19@smu.edu.cn

Q. Fu, Z. Wu, Y. Xiong, L. Zheng
Department of Laboratory Medicine
Nanfang Hospital
Southern Medical University
510515 Guangzhou, China
Email: xyuf9002@smu.edu.cn; nfyyzhenglei@smu.edu.cn

F. Hu
Division of Laboratory Medicine
Zhujiang Hospital
Southern Medical University
510282 Guangzhou, China.
Email: hufang19@smu.edu.cn

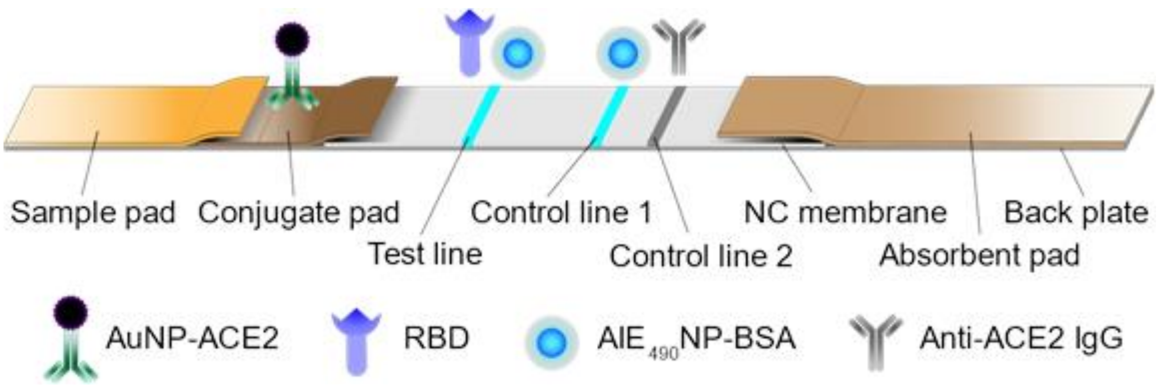

Scheme S1. The structure of the established FQ-LFIA test strip product.

Reagents and solution

Recombined human ACE2 protein and SARS-CoV-2 spike protein (RBD) were purchased from GenScript Corporation (Piscataway, NJ, USA). The anti-ACE2 polyclonal antibody was purchased from SAB LLC (Greenbelt, MD, USA). The carboxyl-modified PS nanoparticles with hydrodynamic diameters of 200 or 300 nm were purchased from Thermo Fisher Scientific (Waltham, MA, USA). Sodium dodecyl sulfate (SDS), bovine serum albumin (BSA), sucrose, trehalose, polyvinyl pyrrolidone (PVP), polyvinyl alcohol (PVA), casein-Na, triton-100, 4-morpholineethanesulfonic acid (MES), 1-ethyl-3-(3-dimethylaminopropyl) carbodiimide hydrochloride (EDC), and N-hydroxysulfosuccinimide (sulfo-NHS) were purchased from Sigma-Aldrich (St. Louis, MO, USA). Nitrocellulose (NC) membrane was purchased from Sartorius AG (Göttingen, Germany). Absorbent paper, fiberglass paper, 0.22 μm syringe filter, and 10 kDa microsep Centrifugal Filters were obtained from Merck Millipore (Bedford, MA, USA). The commercial ELISA kit was purchased from the Cayman Chemical Company (Ann Arbor, MI, USA). All organic solvents used in the experiment were purchased from GHTECH (Guangdong, China).

All solution buffers were made up using ultrapure water prepared from a Milli-Q water purification system (Millipore, Bedford, MA, USA) and the pH was determined using a PB-10 pH meter (Sartorius AG, Göttingen, Germany). The solutions used during this research were as follow: coating buffer (10 mmol/L $Na_2HPO_4{\cdot}12H_2O$, 0.1 % NaN_3 (*w/v*), 0.9 % NaCl (*w/v*), and 0.3 % Trehalose (*w/v*), pH 7.4); activating buffer (25 mmol/L MES, pH 6.1); binding buffer (25 mmol/L phosphate buffer, pH 7.0); washing buffer (25 mmol/L Tris, 0.05 % Proclin-300 (*v/v*), 0.2 % Tween-20 (*v/v*), and 0.9 % NaCl (*w/v*), pH 7.8); blocking buffer (25 mmol/L phosphate buffer, 5 % BSA (*w/v*), pH 7.4); labeled antibody storage buffer (25 mmol/L Tris, 5 % BSA (*w/v*), 1% Trehalose (*w/v*), 1 % sucrose (*w/v*), 0.9 % NaCl (*w/v*), 0.05 % TWEEN-20 (*v/v*), and 0.05 % Proclin-300 (*v/v*), pH 7.2); labeled antibody dilution buffer

(20 mmol/L Tris, 20 % sucrose (*w/v*), 5 % Trehalose (*w/v*), 1 % BSA (*w/v*), and 0.05 % Proclin-300 (*v/v*), pH 9.0); sample pad treatment buffer (100 mmol/L $Na_2B_4O_7·10H_2O$, 1 % PVP (*w/v*), 0.2 % Casein-Na (*w/v*), 0.1 % NaN_3 (*w/v*), and 6 % TritonX-100 (*v/v*)); conjugate pad treatment buffer (50 mmol/L $Na_2HPO_4·12H_2O$, 0.5 % BSA (*w/v*), 0.5 % PVA (*w/v*), and 1 % TritonX-100 (*v/v*), pH 7.4); sample buffer (10 mmol/L $Na_2HPO_4·12H_2O$, 1 % BSA (*w/v*) and 0.9 % NaCl (*w/v*), pH 7.4). After being filtered with the 0.22 μm syringe filter, all solution buffers were stored at 4 °C till use.

Table S1. The anti-SARS-CoV-2 NAbs titers of serum samples (pVNT).

Positive samples		Negative samples	
Sample NO.	IU mL^{-1}	Sample NO.	IU mL^{-1}
SARS-CoV-2-1	30	Pre-SARS-CoV-2-1	<4.173
SARS-CoV-2-2	60	Pre-SARS-CoV-2-2	<4.173
SARS-CoV-2-3	28	Pre-SARS-CoV-2-3	<4.173
SARS-CoV-2-4	28	Pre-SARS-CoV-2-4	<4.173
SARS-CoV-2-5	29	Pre-SARS-CoV-2-5	<4.173
SARS-CoV-2-6	31	Pre-SARS-CoV-2-6	<4.173
SARS-CoV-2-7	31	Pre-SARS-CoV-2-7	<4.173
SARS-CoV-2-8	32	Pre-SARS-CoV-2-8	<4.173
SARS-CoV-2-9	30	Pre-SARS-CoV-2-9	<4.173
SARS-CoV-2-10	48	Pre-SARS-CoV-2-10	<4.173
SARS-CoV-2-11	33	Pre-SARS-CoV-2-11	<4.173
SARS-CoV-2-12	32	Pre-SARS-CoV-2-12	<4.173
SARS-CoV-2-13	133	Pre-SARS-CoV-2-13	<4.173
SARS-CoV-2-14	32	Pre-SARS-CoV-2-14	<4.173
SARS-CoV-2-15	34	Pre-SARS-CoV-2-15	<4.173
SARS-CoV-2-16	61	Pre-SARS-CoV-2-16	<4.173
SARS-CoV-2-17	30	Pre-SARS-CoV-2-17	<4.173
SARS-CoV-2-18	35	Pre-SARS-CoV-2-18	<4.173
SARS-CoV-2-19	42	Pre-SARS-CoV-2-19	<4.173
SARS-CoV-2-20	30	Pre-SARS-CoV-2-20	<4.173
SARS-CoV-2-21	30	Pre-SARS-CoV-2-21	<4.173
SARS-CoV-2-22	108	Pre-SARS-CoV-2-22	<4.173
SARS-CoV-2-23	51	Pre-SARS-CoV-2-23	<4.173
SARS-CoV-2-24	84	Pre-SARS-CoV-2-24	<4.173
SARS-CoV-2-25	40	Pre-SARS-CoV-2-25	<4.173
SARS-CoV-2-26	47	Pre-SARS-CoV-2-26	<4.173
SARS-CoV-2-27	47	Pre-SARS-CoV-2-27	<4.173
SARS-CoV-2-28	51	Pre-SARS-CoV-2-28	<4.173
SARS-CoV-2-29	40	Pre-SARS-CoV-2-29	<4.173

SARS-CoV-2-30	43	Pre-SARS-CoV-2-30	<4.173
SARS-CoV-2-31	48	Pre-SARS-CoV-2-31	<4.173
SARS-CoV-2-32	45	Pre-SARS-CoV-2-32	<4.173
SARS-CoV-2-33	70	Pre-SARS-CoV-2-33	<4.173
SARS-CoV-2-34	69	Pre-SARS-CoV-2-34	<4.173
SARS-CoV-2-35	29	Pre-SARS-CoV-2-35	<4.173
SARS-CoV-2-36	37	Pre-SARS-CoV-2-36	<4.173
SARS-CoV-2-37	69	Pre-SARS-CoV-2-37	<4.173
SARS-CoV-2-38	28	Pre-SARS-CoV-2-38	<4.173
SARS-CoV-2-39	81	Pre-SARS-CoV-2-39	<4.173
SARS-CoV-2-40	88	Pre-SARS-CoV-2-40	<4.173
SARS-CoV-2-41	56	Pre-SARS-CoV-2-41	<4.173
SARS-CoV-2-42	113	Pre-SARS-CoV-2-42	<4.173
SARS-CoV-2-43	61	Pre-SARS-CoV-2-43	<4.173
SARS-CoV-2-44	57	Pre-SARS-CoV-2-44	<4.173
SARS-CoV-2-45	42	Pre-SARS-CoV-2-45	<4.173
SARS-CoV-2-46	42	Pre-SARS-CoV-2-46	<4.173
SARS-CoV-2-47	37	Pre-SARS-CoV-2-47	<4.173
SARS-CoV-2-48	62	Pre-SARS-CoV-2-48	<4.173
SARS-CoV-2-49	28	Pre-SARS-CoV-2-49	<4.173
SARS-CoV-2-50	35	Pre-SARS-CoV-2-50	<4.173
SARS-CoV-2-51	102		
SARS-CoV-2-52	55		
SARS-CoV-2-53	52		
SARS-CoV-2-54	43		
SARS-CoV-2-55	69		
SARS-CoV-2-56	28		
SARS-CoV-2-57	46		
SARS-CoV-2-58	77		
SARS-CoV-2-59	87		
SARS-CoV-2-60	31		
SARS-CoV-2-61	32		
SARS-CoV-2-62	163		
SARS-CoV-2-63	52		
SARS-CoV-2-64	31		
SARS-CoV-2-65	33		
SARS-CoV-2-66	37		
SARS-CoV-2-67	38		
SARS-CoV-2-68	151		
SARS-CoV-2-69	43		
SARS-CoV-2-70	37		
SARS-CoV-2-71	50		
SARS-CoV-2-72	66		
SARS-CoV-2-73	51		

SARS-CoV-2-74	70
SARS-CoV-2-75	128
SARS-CoV-2-76	32
SARS-CoV-2-77	42
SARS-CoV-2-78	42
SARS-CoV-2-79	29
SARS-CoV-2-80	42
SARS-CoV-2-81	44
SARS-CoV-2-82	34
SARS-CoV-2-83	69
SARS-CoV-2-84	478
SARS-CoV-2-85	345
SARS-CoV-2-86	421
SARS-CoV-2-87	360
SARS-CoV-2-88	580
SARS-CoV-2-89	491
SARS-CoV-2-90	760
SARS-CoV-2-91	307
SARS-CoV-2-92	248
SARS-CoV-2-93	319
SARS-CoV-2-94	352
SARS-CoV-2-95	288
SARS-CoV-2-96	260
SARS-CoV-2-97	597
SARS-CoV-2-98	407
SARS-CoV-2-99	327
SARS-CoV-2-100	265
SARS-CoV-2-101	470
SARS-CoV-2-102	615
SARS-CoV-2-103	445

Table S2. The background information of the serum samples.

Sample NO.	Days after the last injection	Gender	Age	Vaccine	Manufacturer	Type of vaccine	Jabs
SARS-CoV-2-1	26	Male	34	COVILO	Beijing Institute of Biological Products Co., Ltd.	Inactivated vaccine	3
SARS-CoV-2-2	32	Female	29	COVILO	Beijing Institute of Biological Products Co., Ltd.	Inactivated vaccine	3
SARS-CoV-2-3	36	Female	26	COVILO	Beijing Institute of Biological Products Co., Ltd.	Inactivated vaccine	3
SARS-CoV-2-4	22	Female	25	COVILO	Beijing Institute of Biological Products Co., Ltd.	Inactivated vaccine	3
SARS-CoV-2-5	26	Male	35	COVILO	Beijing Institute of Biological Products Co., Ltd.	Inactivated vaccine	3
SARS-CoV-2-6	43	Female	30	COVILO	Beijing Institute of Biological Products Co., Ltd.	Inactivated vaccine	3
SARS-CoV-2-7	35	Female	27	COVILO	Beijing Institute of Biological Products Co., Ltd.	Inactivated vaccine	3
SARS-CoV-2-8	22	Female	42	COVILO	Beijing Institute of Biological Products Co., Ltd.	Inactivated vaccine	3

SARS-CoV-2-9	33	Female	33	COVILO	Beijing Institute of Biological Products Co., Ltd.	Inactivated vaccine	3
SARS-CoV-2-10	31	Male	37	COVILO	Beijing Institute of Biological Products Co., Ltd.	Inactivated vaccine	3
SARS-CoV-2-11	37	Male	25	COVILO	Beijing Institute of Biological Products Co., Ltd.	Inactivated vaccine	3
SARS-CoV-2-12	34	Male	25	COVILO	Beijing Institute of Biological Products Co., Ltd.	Inactivated vaccine	3
SARS-CoV-2-13	37	Male	34	COVILO	Beijing Institute of Biological Products Co., Ltd.	Inactivated vaccine	3
SARS-CoV-2-14	40	Female	29	COVILO	Beijing Institute of Biological Products Co., Ltd.	Inactivated vaccine	3
SARS-CoV-2-15	22	Female	38	COVILO	Beijing Institute of Biological Products Co., Ltd.	Inactivated vaccine	3
SARS-CoV-2-16	22	Male	31	COVILO	Beijing Institute of Biological Products Co., Ltd.	Inactivated vaccine	3
SARS-CoV-2-17	23	Female	41	COVILO	Beijing Institute of Biological Products Co., Ltd.	Inactivated vaccine	3
SARS-CoV-2-18	29	Male	27	COVILO	Beijing Institute of Biological Products Co., Ltd.	Inactivated vaccine	3
SARS-CoV-2-19	25	Male	36	COVILO	Beijing Institute of Biological Products Co., Ltd.	Inactivated vaccine	3
SARS-CoV-2-20	26	Female	25	COVILO	Beijing Institute of Biological Products Co., Ltd.	Inactivated vaccine	3
SARS-CoV-2-21	38	Female	26	COVILO	Beijing Institute of Biological Products Co., Ltd.	Inactivated vaccine	3
SARS-CoV-2-22	23	Male	42	COVILO	Beijing Institute of Biological Products Co., Ltd.	Inactivated vaccine	3
SARS-CoV-2-23	25	Male	34	COVILO	Beijing Institute of Biological Products Co., Ltd.	Inactivated vaccine	3
SARS-CoV-2-24	33	Female	42	COVILO	Beijing Institute of Biological Products Co., Ltd.	Inactivated vaccine	3
SARS-CoV-2-25	37	Female	25	COVILO	Beijing Institute of Biological Products Co., Ltd.	Inactivated vaccine	3
SARS-CoV-2-26	43	Male	29	COVILO	Beijing Institute of Biological Products Co., Ltd.	Inactivated vaccine	3
SARS-CoV-2-27	22	Female	42	COVILO	Beijing Institute of Biological Products Co., Ltd.	Inactivated vaccine	3
SARS-CoV-2-28	21	Male	34	COVILO	Beijing Institute of Biological Products Co., Ltd.	Inactivated vaccine	3
SARS-CoV-2-29	29	Female	36	COVILO	Beijing Institute of Biological Products Co., Ltd.	Inactivated vaccine	3
SARS-CoV-2-30	44	Male	24	COVILO	Beijing Institute of Biological Products Co., Ltd.	Inactivated vaccine	3
SARS-CoV-2-31	41	Female	30	COVILO	Beijing Institute of Biological Products Co., Ltd.	Inactivated vaccine	3
SARS-CoV-2-32	28	Female	25	COVILO	Beijing Institute of Biological Products Co., Ltd.	Inactivated vaccine	3
SARS-CoV-2-33	22	Female	38	COVILO	Beijing Institute of Biological Products Co., Ltd.	Inactivated vaccine	3
SARS-CoV-2-34	26	Male	24	COVILO	Beijing Institute of Biological Products Co., Ltd.	Inactivated vaccine	3
SARS-CoV-2-35	35	Female	34	COVILO	Beijing Institute of Biological Products Co., Ltd.	Inactivated vaccine	3
SARS-CoV-2-36	45	Female	37	COVILO	Beijing Institute of Biological Products Co., Ltd.	Inactivated vaccine	3
SARS-CoV-2-37	41	Female	28	COVILO	Beijing Institute of Biological Products Co., Ltd.	Inactivated vaccine	3
SARS-CoV-2-38	35	Male	39	COVILO	Beijing Institute of Biological Products Co., Ltd.	Inactivated vaccine	3
SARS-CoV-2-39	32	Female	25	COVILO	Beijing Institute of Biological Products Co., Ltd.	Inactivated vaccine	3
SARS-CoV-2-40	35	Female	30	COVILO	Beijing Institute of Biological Products Co., Ltd.	Inactivated vaccine	3
SARS-CoV-2-41	35	Female	37	COVILO	Beijing Institute of Biological Products Co., Ltd.	Inactivated vaccine	3
SARS-CoV-2-42	43	Female	30	COVILO	Beijing Institute of Biological Products Co., Ltd.	Inactivated vaccine	3
SARS-CoV-2-43	32	Male	24	COVILO	Beijing Institute of Biological Products Co., Ltd.	Inactivated vaccine	3
SARS-CoV-2-44	33	Male	33	COVILO	Beijing Institute of Biological Products Co., Ltd.	Inactivated vaccine	3
SARS-CoV-2-45	41	Female	33	COVILO	Beijing Institute of Biological Products Co., Ltd.	Inactivated vaccine	3
SARS-CoV-2-46	43	Female	40	COVILO	Beijing Institute of Biological Products Co., Ltd.	Inactivated vaccine	3

SARS-CoV-2-47	35	Male	28	COVILO	Beijing Institute of Biological Products Co., Ltd.	Inactivated vaccine	3
SARS-CoV-2-48	35	Female	33	COVILO	Beijing Institute of Biological Products Co., Ltd.	Inactivated vaccine	3
SARS-CoV-2-49	32	Female	29	COVILO	Beijing Institute of Biological Products Co., Ltd.	Inactivated vaccine	3
SARS-CoV-2-50	25	Male	42	COVILO	Beijing Institute of Biological Products Co., Ltd.	Inactivated vaccine	3
SARS-CoV-2-51	27	Male	40	COVILO	Beijing Institute of Biological Products Co., Ltd.	Inactivated vaccine	3
SARS-CoV-2-52	34	Female	30	COVILO	Beijing Institute of Biological Products Co., Ltd.	Inactivated vaccine	3
SARS-CoV-2-53	36	Male	41	COVILO	Beijing Institute of Biological Products Co., Ltd.	Inactivated vaccine	3
SARS-CoV-2-54	21	Female	28	COVILO	Beijing Institute of Biological Products Co., Ltd.	Inactivated vaccine	3
SARS-CoV-2-55	28	Female	36	COVILO	Beijing Institute of Biological Products Co., Ltd.	Inactivated vaccine	3
SARS-CoV-2-56	21	Female	39	COVILO	Beijing Institute of Biological Products Co., Ltd.	Inactivated vaccine	3
SARS-CoV-2-57	30	Male	27	COVILO	Beijing Institute of Biological Products Co., Ltd.	Inactivated vaccine	3
SARS-CoV-2-58	24	Female	41	COVILO	Beijing Institute of Biological Products Co., Ltd.	Inactivated vaccine	3
SARS-CoV-2-59	38	Male	37	COVILO	Beijing Institute of Biological Products Co., Ltd.	Inactivated vaccine	3
SARS-CoV-2-60	23	Female	32	COVILO	Beijing Institute of Biological Products Co., Ltd.	Inactivated vaccine	3
SARS-CoV-2-61	38	Male	25	COVILO	Beijing Institute of Biological Products Co., Ltd.	Inactivated vaccine	3
SARS-CoV-2-62	27	Male	41	COVILO	Beijing Institute of Biological Products Co., Ltd.	Inactivated vaccine	3
SARS-CoV-2-63	24	Male	29	COVILO	Beijing Institute of Biological Products Co., Ltd.	Inactivated vaccine	3
SARS-CoV-2-64	35	Male	33	COVILO	Beijing Institute of Biological Products Co., Ltd.	Inactivated vaccine	3
SARS-CoV-2-65	21	Male	33	COVILO	Beijing Institute of Biological Products Co., Ltd.	Inactivated vaccine	3
SARS-CoV-2-66	31	Female	35	CoronaVac	Sinovac Life Sciences Co., Ltd.	Inactivated vaccine	3
SARS-CoV-2-67	40	Male	26	CoronaVac	Sinovac Life Sciences Co., Ltd.	Inactivated vaccine	3
SARS-CoV-2-68	35	Male	29	CoronaVac	Sinovac Life Sciences Co., Ltd.	Inactivated vaccine	3
SARS-CoV-2-69	45	Female	28	CoronaVac	Sinovac Life Sciences Co., Ltd.	Inactivated vaccine	3
SARS-CoV-2-70	31	Male	41	CoronaVac	Sinovac Life Sciences Co., Ltd.	Inactivated vaccine	3
SARS-CoV-2-71	43	Female	29	CoronaVac	Sinovac Life Sciences Co., Ltd.	Inactivated vaccine	3
SARS-CoV-2-72	42	Male	43	CoronaVac	Sinovac Life Sciences Co., Ltd.	Inactivated vaccine	3
SARS-CoV-2-73	45	Female	31	CoronaVac	Sinovac Life Sciences Co., Ltd.	Inactivated vaccine	3
SARS-CoV-2-74	33	Male	41	CoronaVac	Sinovac Life Sciences Co., Ltd.	Inactivated vaccine	3
SARS-CoV-2-75	45	Female	32	CoronaVac	Sinovac Life Sciences Co., Ltd.	Inactivated vaccine	3
SARS-CoV-2-76	25	Female	37	CoronaVac	Sinovac Life Sciences Co., Ltd.	Inactivated vaccine	3
SARS-CoV-2-77	22	Female	25	CoronaVac	Sinovac Life Sciences Co., Ltd.	Inactivated vaccine	3
SARS-CoV-2-78	28	Male	30	CoronaVac	Sinovac Life Sciences Co., Ltd.	Inactivated vaccine	3
SARS-CoV-2-79	29	Male	27	CoronaVac	Sinovac Life Sciences Co., Ltd.	Inactivated vaccine	3
SARS-CoV-2-80	41	Female	33	CoronaVac	Sinovac Life Sciences Co., Ltd.	Inactivated vaccine	3
SARS-CoV-2-81	45	Female	27	CoronaVac	Sinovac Life Sciences Co., Ltd.	Inactivated vaccine	3
SARS-CoV-2-82	26	Male	38	CoronaVac	Sinovac Life Sciences Co., Ltd.	Inactivated vaccine	3
SARS-CoV-2-83	21	Male	28	CoronaVac	Sinovac Life Sciences Co., Ltd.	Inactivated vaccine	3
SARS-CoV-2-84	25	Male	42	CoronaVac	Sinovac Life Sciences Co., Ltd.	Inactivated vaccine	3

SARS-CoV-2-85	14	Female	31	ZF2001	Anhui Zhifei Longcom Biopharmaceutical Co., Ltd.	Recombinant vaccine	3
SARS-CoV-2-86	26	Male	24	ZF2002	Anhui Zhifei Longcom Biopharmaceutical Co., Ltd.	Recombinant vaccine	3
SARS-CoV-2-87	21	Male	41	ZF2003	Anhui Zhifei Longcom Biopharmaceutical Co., Ltd.	Recombinant vaccine	3
SARS-CoV-2-88	19	Female	30	ZF2004	Anhui Zhifei Longcom Biopharmaceutical Co., Ltd.	Recombinant vaccine	3
SARS-CoV-2-89	15	Female	29	ZF2005	Anhui Zhifei Longcom Biopharmaceutical Co., Ltd.	Recombinant vaccine	3
SARS-CoV-2-90	22	Female	38	ZF2006	Anhui Zhifei Longcom Biopharmaceutical Co., Ltd.	Recombinant vaccine	3
SARS-CoV-2-91	16	Female	38	ZF2007	Anhui Zhifei Longcom Biopharmaceutical Co., Ltd.	Recombinant vaccine	3
SARS-CoV-2-92	19	Male	31	ZF2008	Anhui Zhifei Longcom Biopharmaceutical Co., Ltd.	Recombinant vaccine	3
SARS-CoV-2-93	23	Female	36	ZF2009	Anhui Zhifei Longcom Biopharmaceutical Co., Ltd.	Recombinant vaccine	3
SARS-CoV-2-94	18	Male	36	ZF2010	Anhui Zhifei Longcom Biopharmaceutical Co., Ltd.	Recombinant vaccine	3
SARS-CoV-2-95	15	Female	29	ZF2011	Anhui Zhifei Longcom Biopharmaceutical Co., Ltd.	Recombinant vaccine	3
SARS-CoV-2-96	15	Male	43	ZF2012	Anhui Zhifei Longcom Biopharmaceutical Co., Ltd.	Recombinant vaccine	3
SARS-CoV-2-97	19	Male	43	ZF2013	Anhui Zhifei Longcom Biopharmaceutical Co., Ltd.	Recombinant vaccine	3
SARS-CoV-2-98	22	Male	26	ZF2014	Anhui Zhifei Longcom Biopharmaceutical Co., Ltd.	Recombinant vaccine	3
SARS-CoV-2-99	20	Female	35	ZF2015	Anhui Zhifei Longcom Biopharmaceutical Co., Ltd.	Recombinant vaccine	3
SARS-CoV-2-100	16	Female	35	ZF2016	Anhui Zhifei Longcom Biopharmaceutical Co., Ltd.	Recombinant vaccine	3
SARS-CoV-2-101	20	Male	35	ZF2017	Anhui Zhifei Longcom Biopharmaceutical Co., Ltd.	Recombinant vaccine	3
SARS-CoV-2-102	22	Female	25	ZF2018	Anhui Zhifei Longcom Biopharmaceutical Co., Ltd.	Recombinant vaccine	3
SARS-CoV-2-103	16	Male	40	ZF2019	Anhui Zhifei Longcom Biopharmaceutical Co., Ltd.	Recombinant vaccine	3

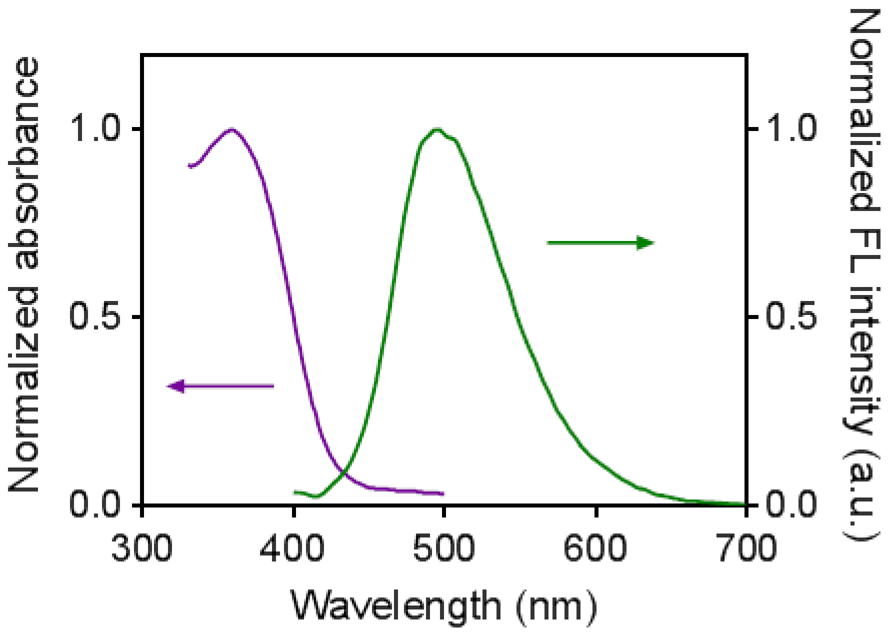

Figure S1. The absorption and fluorescence spectra of AIE_{490}.

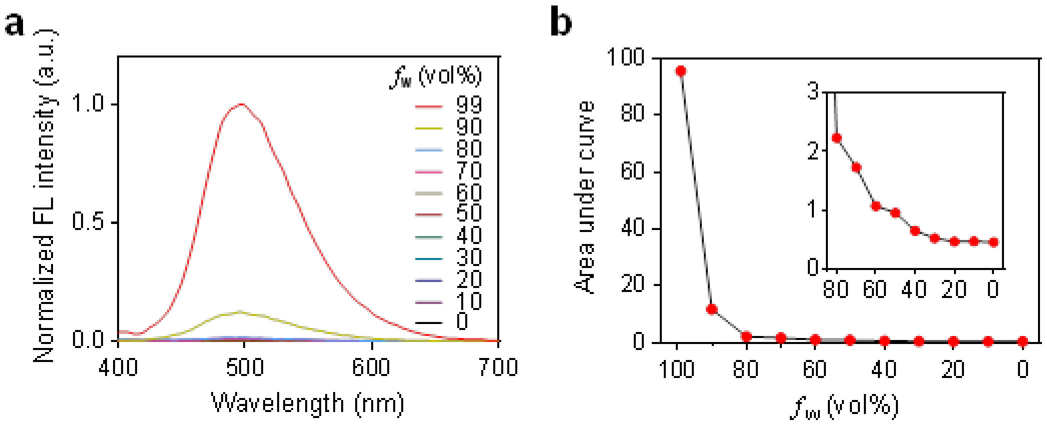

Figure S2. The AIE properties of AIE_{490}. (a) The fluorescence intensity of AIE_{490} in THF and THF/water mixture with different water fractions (f_w). (b) The fluorescence integration of AIE_{490} in THF and THF/water mixture with different water fractions (f_w).

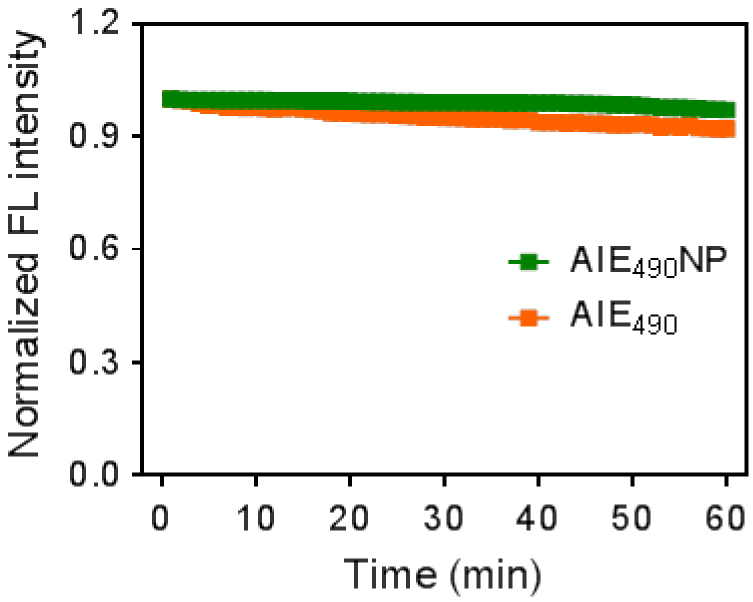

Figure S3. The fluorescence intensity changing trends of AIE_{490} under continuous irradiation of white light (100 mW cm^{-2}).

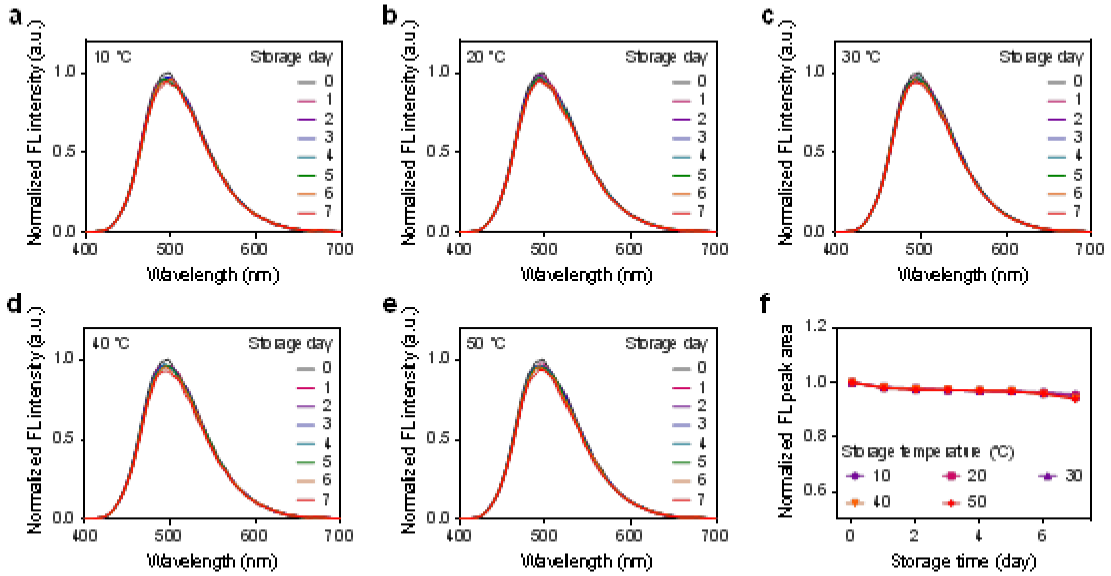

Figure S4. The fluorescence intensity changing of AIE_{490} stored at 10 °C (a), 20 °C (b), 30 °C (c), 40 °C (d), and 50 °C (e). (f) The fluorescence intensity changing trends of AIE_{490} stored at different temperatures.

Table S3. The loss of fluorescence intensity of AIE_{490} and $AIE_{490}NP$ under different temperatures.

Loss of Fluorescence intensity (%)	Storage temperature (°C)				
	10	20	30	40	50
AIE_{490}	4.3	4.9	5.1	5.8	6.1
$AIE_{490}NP$	0.5	0.6	1.1	1.5	2.3

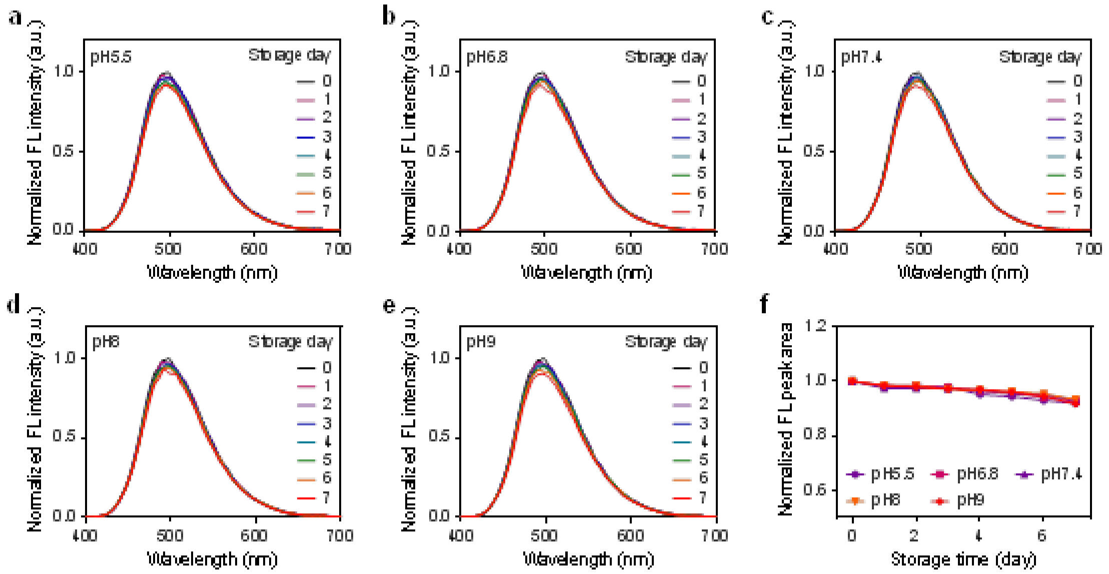

Figure S5. The fluorescence intensity changing of AIE_{490} stored at pH 5.5 (a), pH 6.8 (b), pH 7.4 (c), pH 8 (d), and pH 9 (e). (f) The fluorescence intensity changing trends of AIE_{490} stored at different pH levels.

Table S4. The loss of fluorescence intensity of AIE_{490} and $AIE_{490}NP$ under different pH levels.

Loss of Fluorescence intensity (%)	Storage pH				
	5.5	6.8	7.4	8	9
AIE_{490}	8.0	7.3	6.9	6.6	8.3
$AIE_{490}NP$	4.3	4.6	4.5	4.8	4.9

Calculation of the number of AIE_{490} capsulated in an $AIE_{490}NP$.

First, the absorbance spectra of AIE_{490} (in THF/water, v/v = 1/99) with various concentrations by serial dilution were measured (Figure S6a). The absorbance value at 365 nm exhibited a linear relationship with the concentration of AIE_{490}. The standard curve was obtained by plotting the absorbance value at 365 nm (Y) against the concentration of AIE_{490} (X) as represented by the equation: Y=0.02158*X+0.001525 with a reliable coefficient of determination (R^2=0.9976) (Figure S6b). 1 mg of $AIE_{490}NP$ was freeze-dried and then dissolved in 1 mL of THF to obtain the AIE_{490} encapsulated in $AIE_{490}NP$. Subsequently, 10 μL of supernatant was taken in 990 μL of water and the absorbance of the mixture was measured too (Figure S6c). The mass of the AIE_{490} molecule contained in 1 mg of PS nanoparticles was calculated to be 89.7 μg, which is equivalent to 7.16×10^{16} by the formula of

$N_1=\frac{m}{M}\times n$ (n=6.02×10^{23}/mol, M=753.97 g/mol). Meanwhile, the number of 1 mg of PS was calculated to be 7.08×10^{10} by the formula of $N_2=\frac{m}{\rho\frac{4}{3}\pi r^3}$. The number of AIE_{490} in an AIE_{490}NP was calculated to be 1.01×10^{6} by a formula of $N=\frac{N_1}{N_2}$.

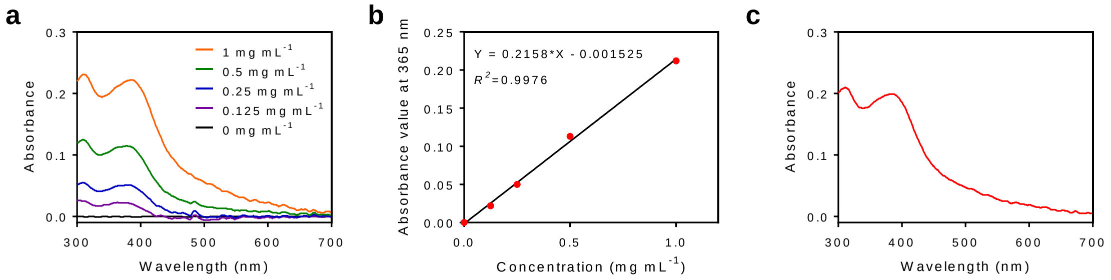

Figure S6. (a) The UV-vis absorption spectra of AIE_{490} with various concentrations in THF/water. (B) The linear relationship between the concentration of AIE_{490} and the absorbance value at 365 nm. (C) UV-vis absorption spectra of AIE_{490}NP dissolved in THF/water. THF/water, v/v = 1/99.

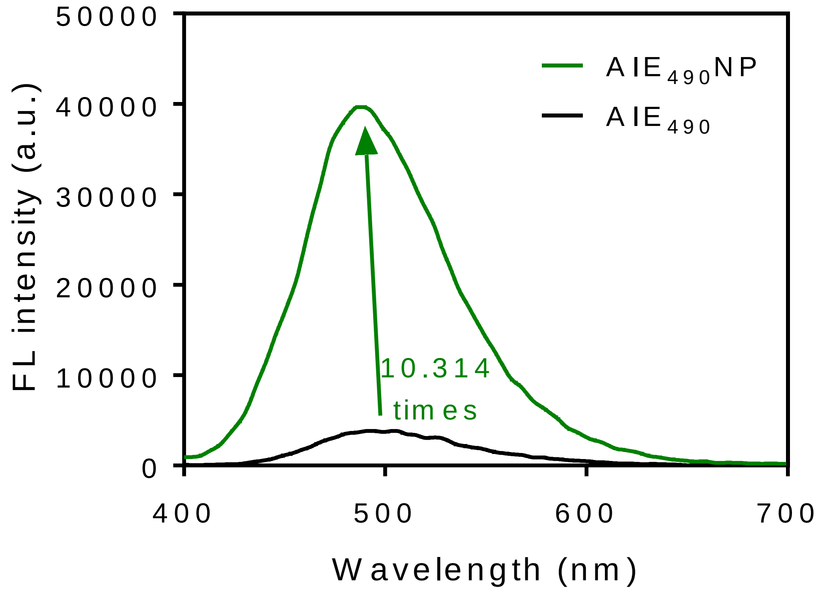

Figure S7. The fluorescence intensity of AIE_{490}NP and AIE_{490} aggregated in water when the concentration of AIE_{490} molecule is the same at 0.897 μg mL^{-1}.

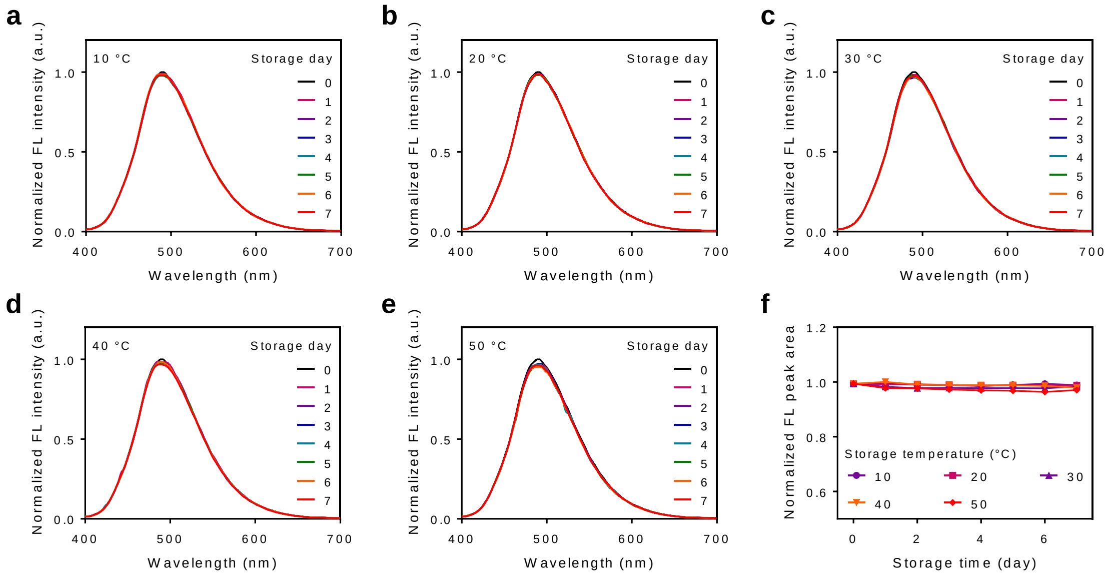

Figure S8. The fluorescence intensity changing of $AIE_{490}NP$ stored at 10 °C (a), 20 °C (b), 30 °C (c), 40 °C (d), and 50 °C (e). (f) The fluorescence intensity changing trends of $AIE_{490}NP$ stored at different temperatures.

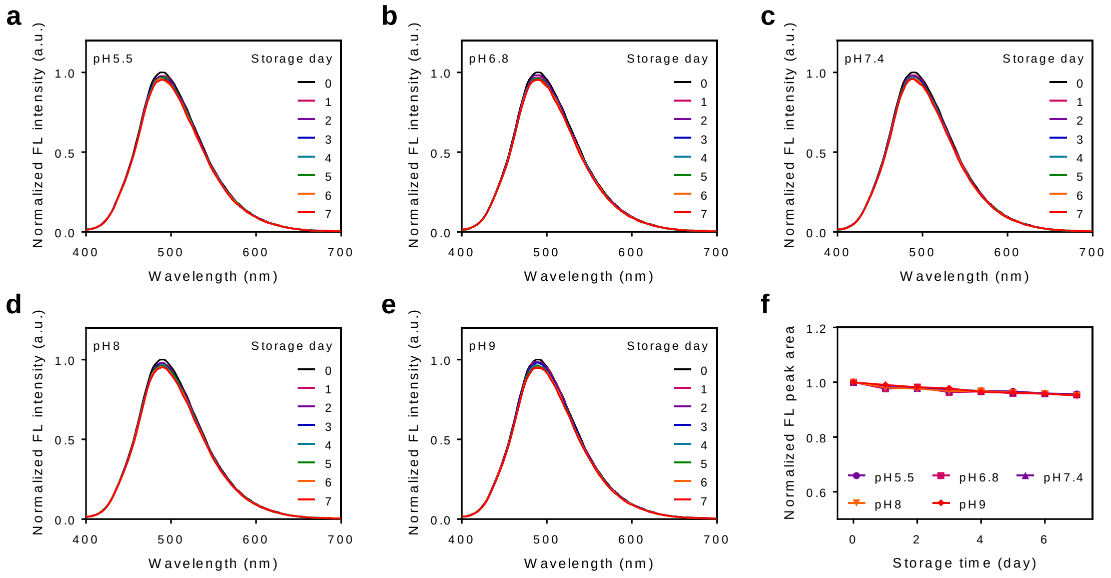

Figure S9. The fluorescence intensity changing of $AIE_{490}NP$ stored at pH 5.5 (a), pH 6.8 (b), pH 7.4 (c), pH 8 (d), and pH 9 (e). (f) The fluorescence intensity changing trends of $AIE_{490}NP$ stored at different pH.

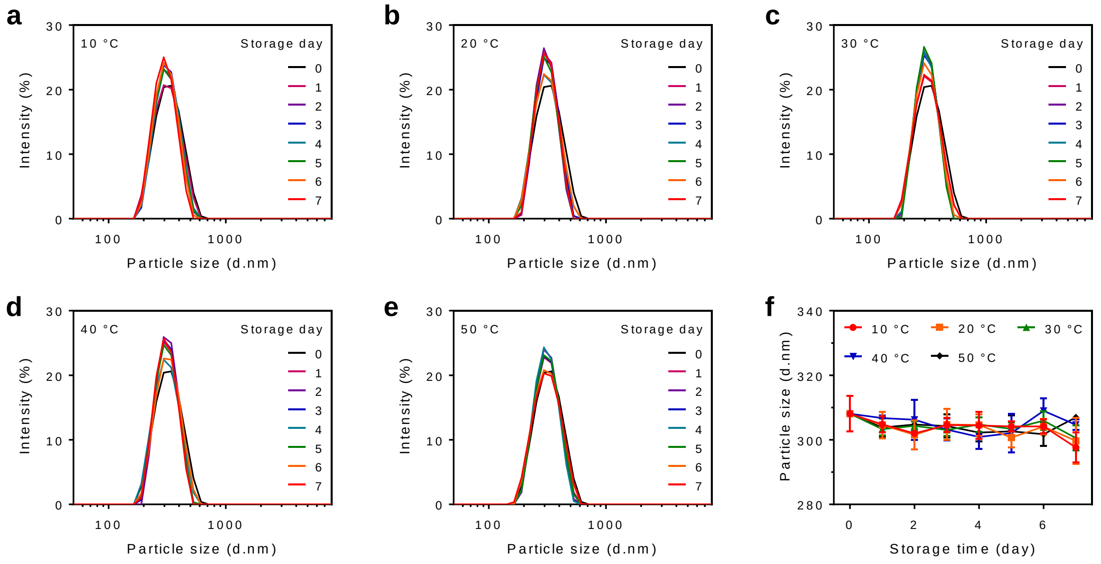

Figure S10. The hydrodynamic diameter changing of AIE$_{490}$NP storing at 10 °C (a), 20 °C (b), 30 °C (c), 40 °C (d), and 50 °C (e). (f) The hydrodynamic diameter changing trends of AIE$_{490}$NP stored at different temperatures.

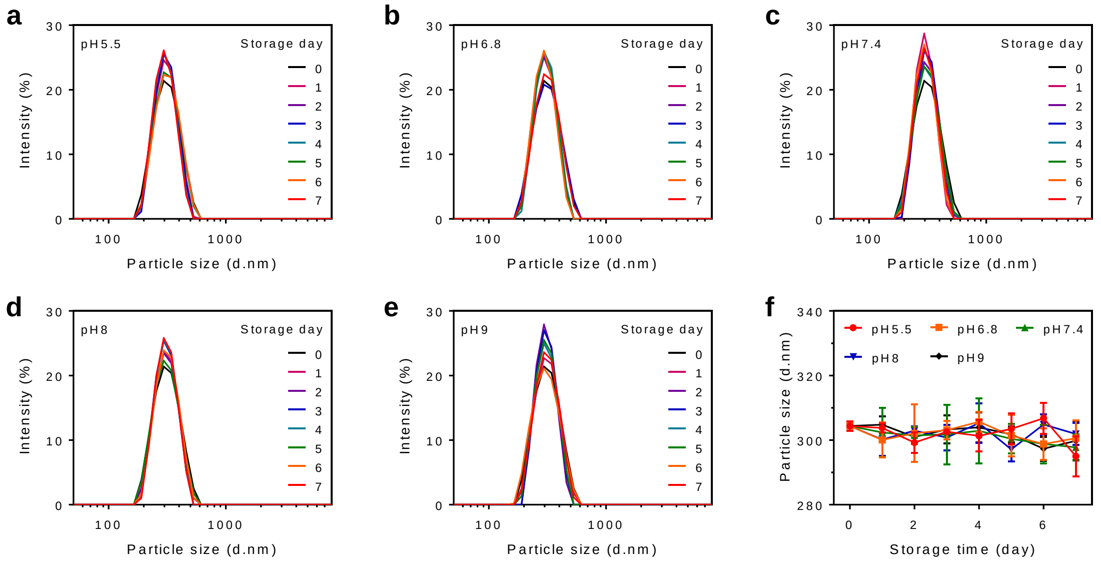

Figure S11. The hydrodynamic diameter changing of AIE$_{490}$NP stored at pH 5.5 (a), pH 6.8 (b), pH 7.4 (c), pH 8 (d), and pH 9 (e). (f) The hydrodynamic diameter changing trends of AIE$_{490}$NP stored at different pH levels.

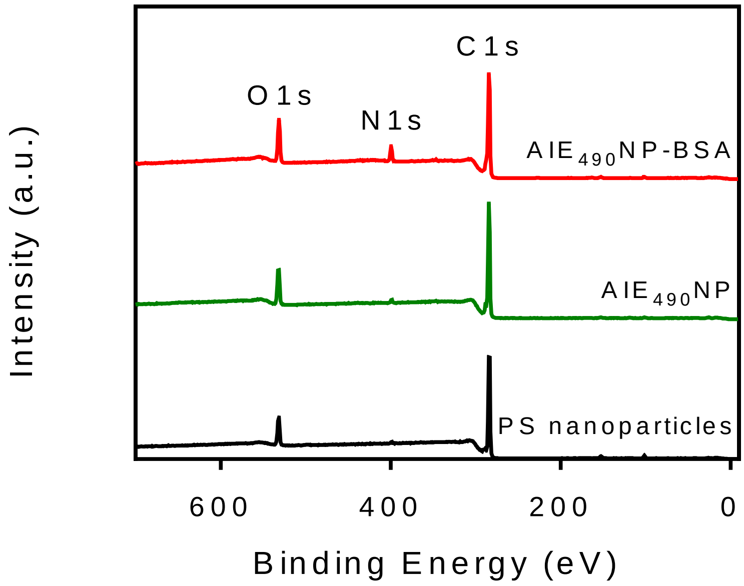

Figure S12. The full range XPS spectra of PS nanoparticles, AIE$_{490}$NP, and AIE$_{490}$NP-BSA.

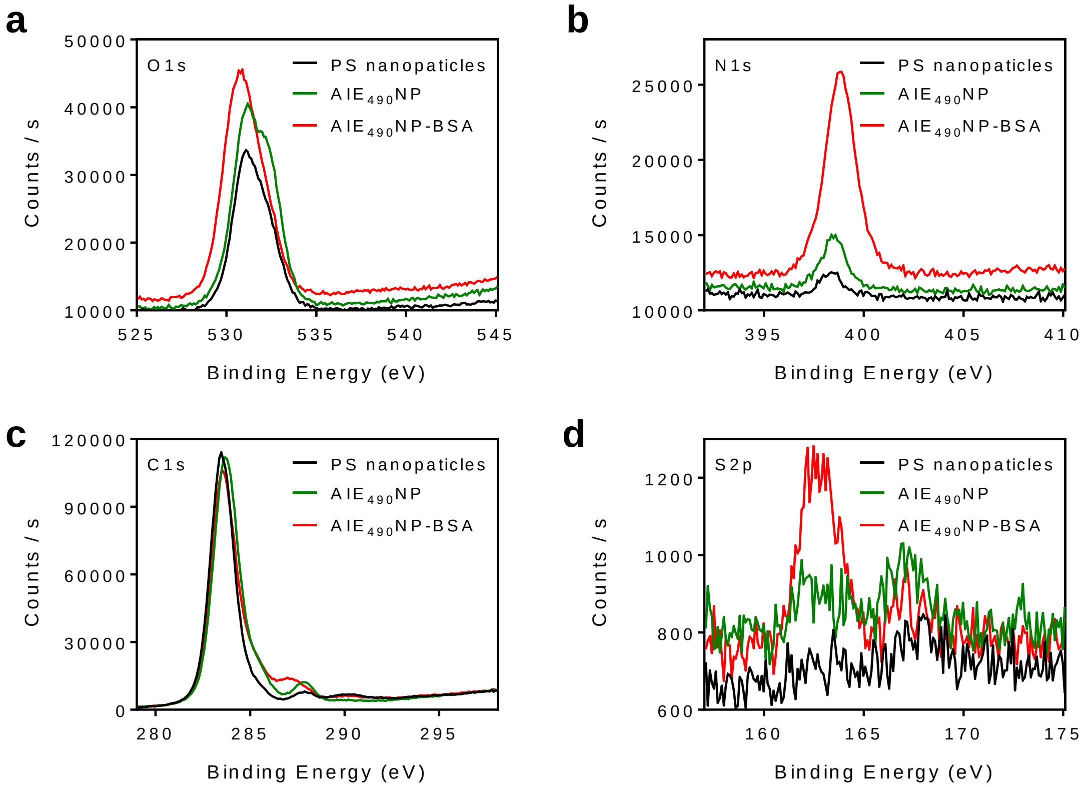

Figure S13. The O1s (a), N1s (b), C1s (c), and S2p (d) spectra of PS nanoparticles, AIE$_{490}$NP, and AIE$_{490}$NP-BSA.

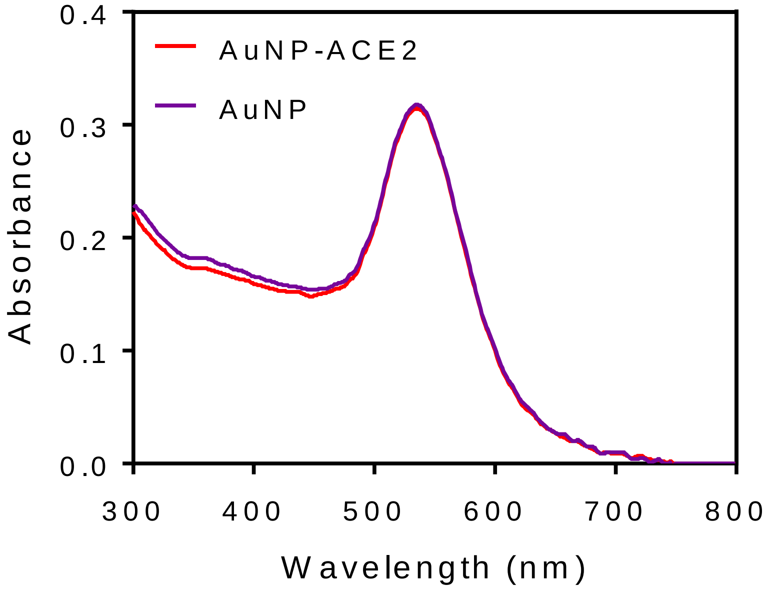

Figure S14. The absorption of AuNP and AuNP-ACE2.

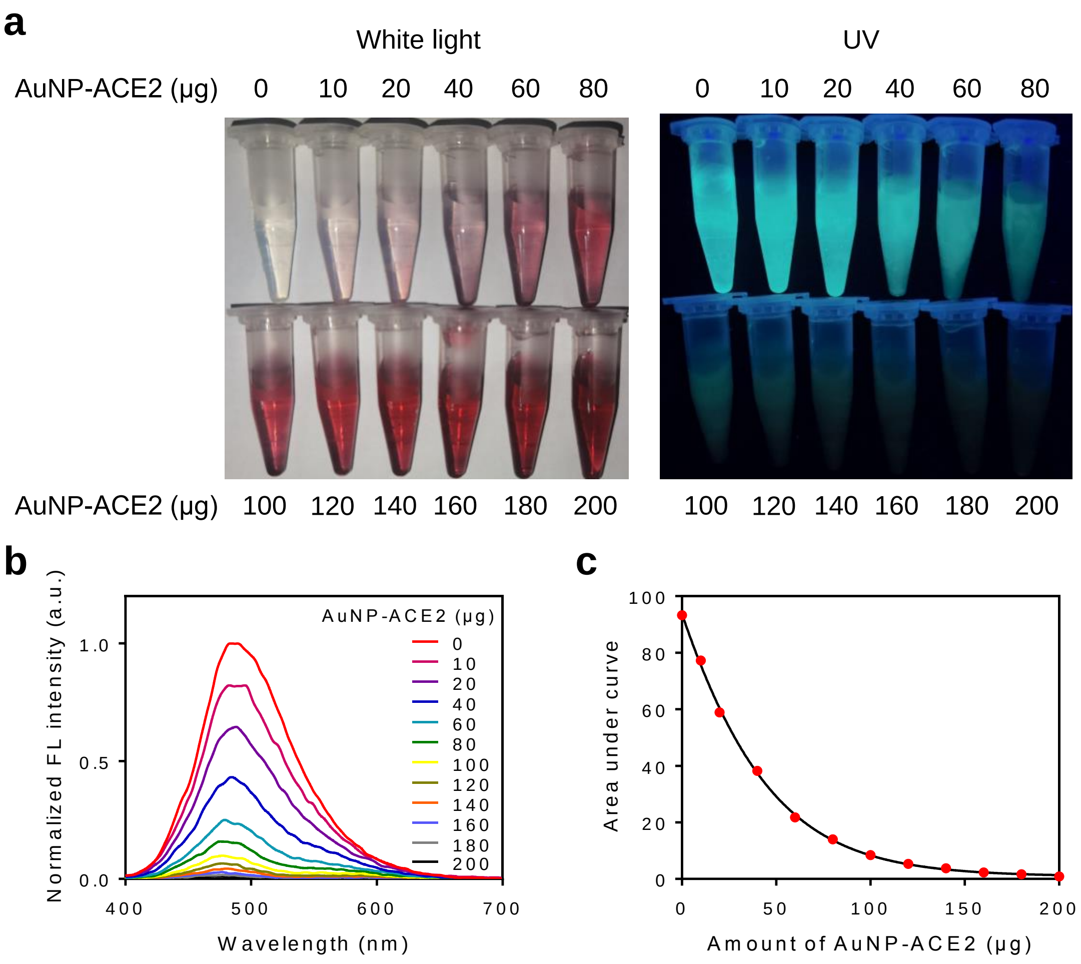

Figure S15. The fluorescence of AIE$_{490}$NP-BSA mixed with different amounts of AuNP-ACE2. (a) The images of 100 μg AIE$_{490}$NP-BSA diluted in 1 mL water containing different amounts (0-200 μg) of AuNP-ACE2 under white light (left) or UV light (right, λ_{ex}=365 nm). (b) The fluorescence intensities of the AIE$_{490}$NP-BSA/AuNP-ACE2 mixture in (a). (c) The area under the curve of the fluorescence intensities in (b).

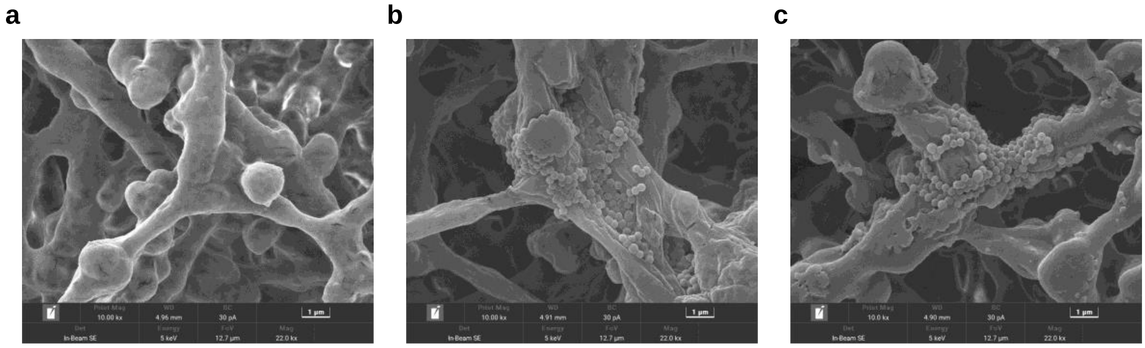

Figure S16. The SEM images of the uncoated NC membrane (a) and the coated test (b) or control line (c) position on the NC membrane.

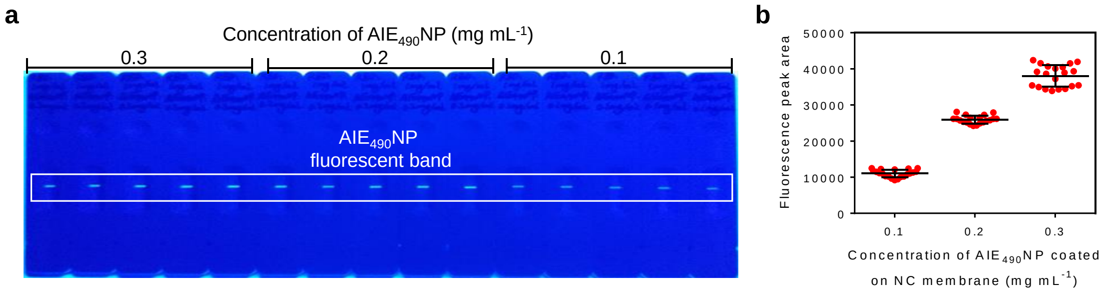

Figure S17. The strips with different concentrations of AIE$_{490}$NP coating on the NC membrane. (a) The picture of the FQ-LFIA strips with test line coated by RBD with a concentration of 1 mg mL^{-1} and AIE$_{490}$NP-BSA with concentrations of 0.1, 0.2, or 0.3 mg mL^{-1}. (b) The fluorescence peak area of the test line in (a) obtained by a portable fluorescence reader.

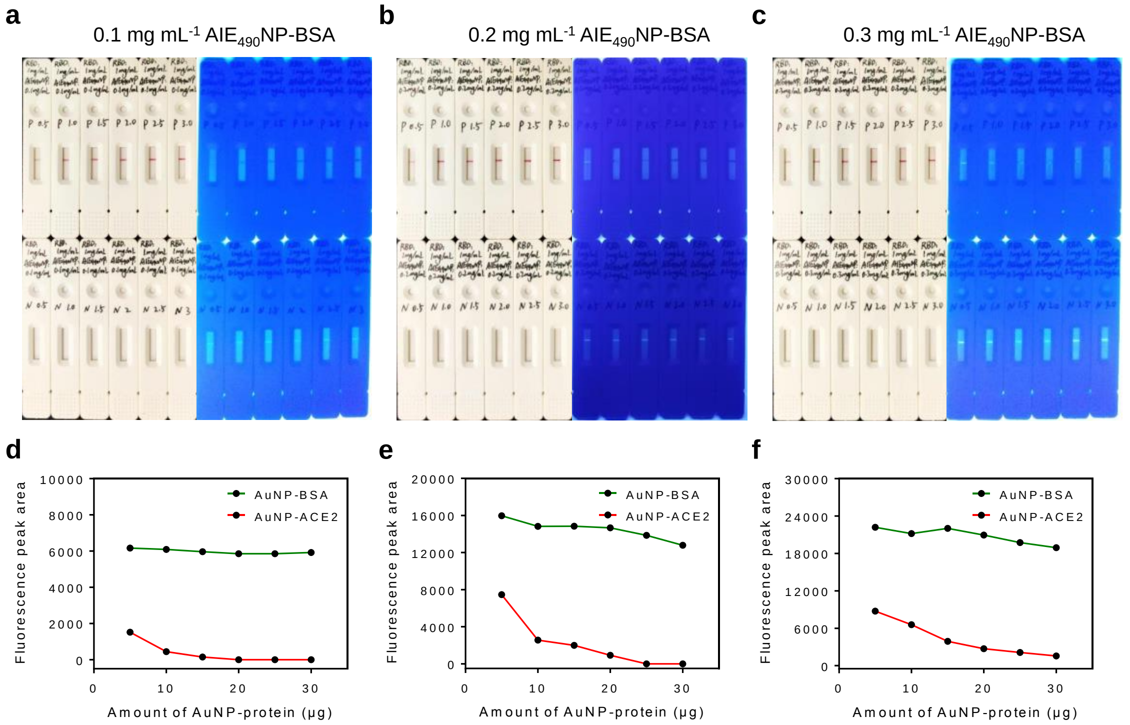

Figure S18. The optimization of AIE_{490}NP-BSA concentration coated on the NC membrane and AuNP-ACE2 amount immobilized on the conjugate pad. The pictures (left: under white light, right: under UV light with λ_{ex} = 365 nm) of the FQ-LFIA strips after detection, different concentrations, 0.1 (a), 0.2 (b), and 0.3 mg mL^{-1} (c) of AIE_{490}NP-BSA were coated at the test line to provide fluorescence signal, and different amounts (5-30 μg) of AuNP-ACE2 (upper) or AuNP-BSA (down) were immobilized on the conjugate pad as experimental group and control group, respectively. (d-f) The fluorescence peak area of the test line in (a-c).

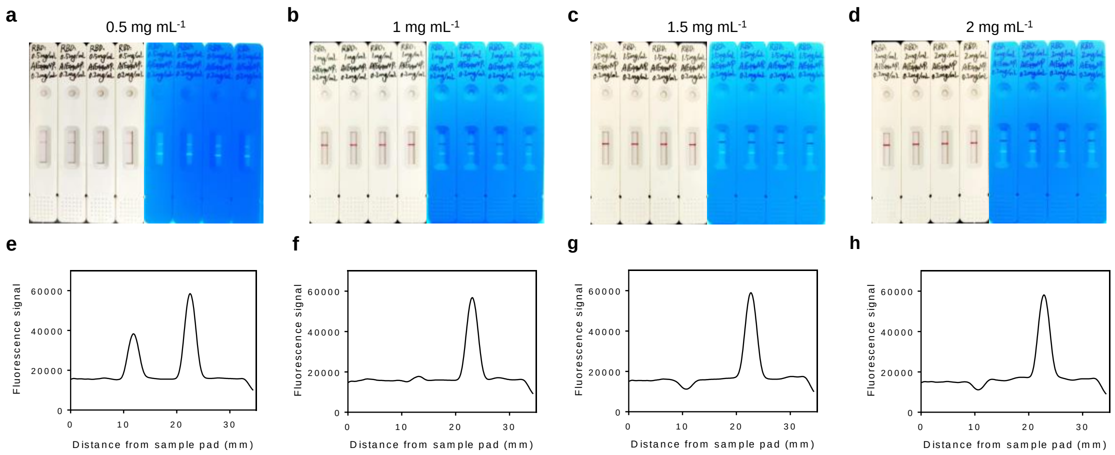

Figure S19. The optimization of RBD concentration coated on the test line. The pictures (a-d, left: under white light, right: under UV light with λ_{ex} = 365 nm) and fluorescence readout

curve (e-f) of test strips used to detect sample buffer; the test strips were coated with different concentrations (0.5, 1, 1.5, and 2 mg mL^{-1}) of RBD on the test line.

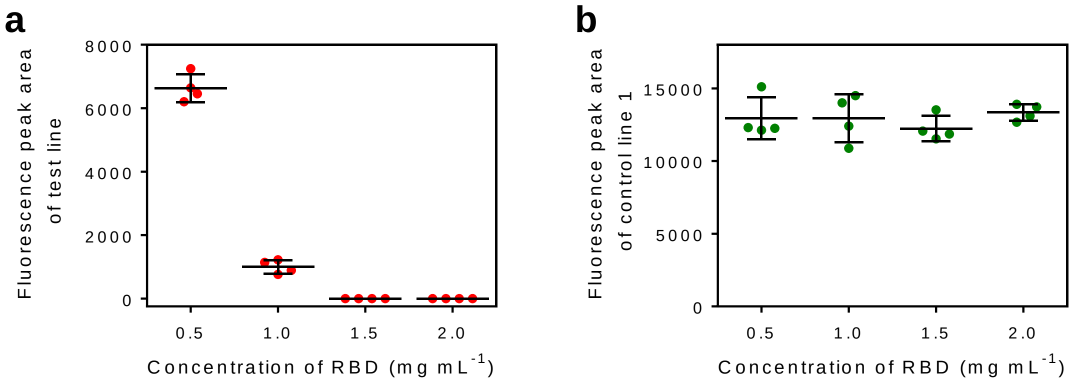

Figure S20. The fluorescence peak area of the test line (a) and control line 1 (b) of test strips used to detect sample buffer which were coated with different concentrations (0.5, 1, 1.5, and 2 mg mL^{-1}) of RBD on the test line.

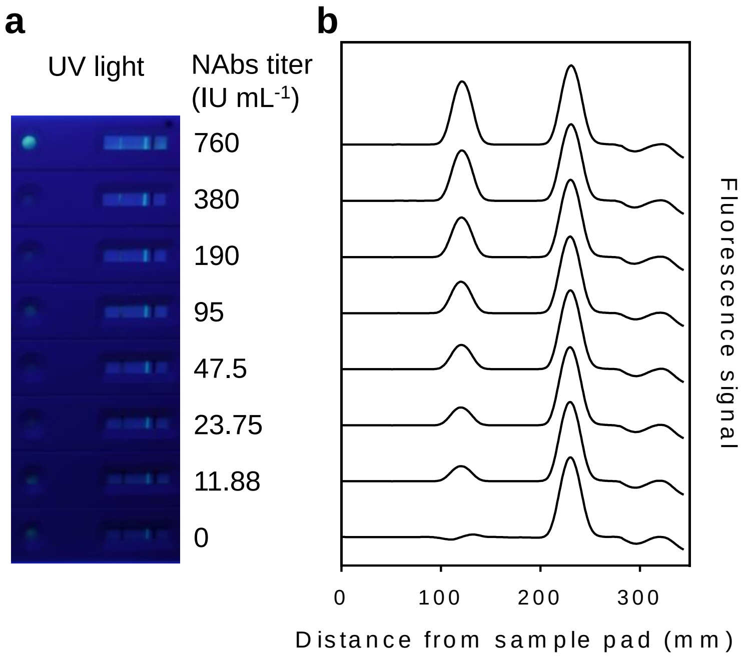

Figure S21. The detection results of calibrators. (a) The picture (under UV light, λ_{ex} = 365 nm) of test strips used for detecting the calibrators with NAbs titers of 760, 380, 190, 95, 47.5, 23.75, and 11.88 IU mL^{-1}. (b) The fluorescence readout curve of test strips in (a).

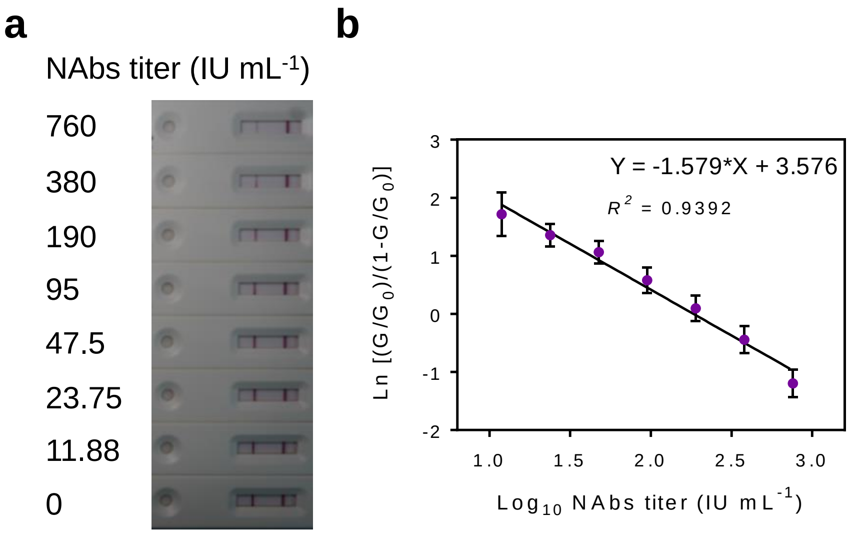

Figure S22. The detection results of calibrators. (a) The pictures of test strips used for detecting the calibrators with NAbs titers of 760, 380, 190, 95, 47.5, 23.75, and 11.88 IU mL^{-1}. (b) The dose-response curve of the detection results through correlating the greyscale and NAbs titer was logit (Y) = -1.579*log (X) + 3.576 (R^2 = 0.9392) and the logit (Y) was calculated as ln [(G/G_0)/(1-G/G_0)], where G is the greyscale of the calibrator and R_0 is that of the sample buffer.

Table S5. The detection results of the 103 positive serum samples and 50 negative serum samples using the pVNT method, the established FQ-LFIA, and the commercial ELISA kit.

Sample NO.	Anti-SARS-CoV-2 NAbs titer (IU mL^{-1})		
	pVNT	FQ-LFIA	ELISA
SARS-CoV-2-1	30.47291	28.77974887	53.02101
SARS-CoV-2-2	60.40052	89.72449032	26.49148
SARS-CoV-2-3	28.35682	29.38238195	38.0043
SARS-CoV-2-4	28.35682	32.55247395	33.99989
SARS-CoV-2-5	28.65912	32.41149025	120.5962
SARS-CoV-2-6	31.37981	23.7479978	-7.546455
SARS-CoV-2-7	31.37981	27.3513017	133.6107
SARS-CoV-2-8	31.98441	27.91630921	114.089
SARS-CoV-2-9	29.86831	39.62169039	67.03667
SARS-CoV-2-10	47.70396	42.65078216	21.98644

SARS-CoV-2-11	33.1936	36.7686767	136.6141
SARS-CoV-2-12	32.28671	21.62584072	143.6219
SARS-CoV-2-13	132.9523	122.5064627	237.7267
SARS-CoV-2-14	31.98441	32.22960627	48.01544
SARS-CoV-2-15	34.1005	36.04250771	148.6274
SARS-CoV-2-16	61.30742	65.45231322	113.5885
SARS-CoV-2-17	30.17061	38.00327287	141.6197
SARS-CoV-2-18	35.0074	40.97811718	108.5829
SARS-CoV-2-19	41.96028	63.60320492	109.0834
SARS-CoV-2-20	29.56602	31.68538584	9.472574
SARS-CoV-2-21	29.56602	32.18422087	27.49264
SARS-CoV-2-22	108.1638	104.4054092	158.6386
SARS-CoV-2-23	51.02925	49.91280764	32.99873
SARS-CoV-2-24	83.67754	59.48753865	43.51039
SARS-CoV-2-25	39.84418	36.46489774	92.06451
SARS-CoV-2-26	47.40166	53.98337092	49.51712
SARS-CoV-2-27	46.79706	53.21805524	10.97426
SARS-CoV-2-28	51.33155	58.5815691	54.52269
SARS-CoV-2-29	39.84418	41.28402192	134.6118
SARS-CoV-2-30	42.86718	46.32442101	143.6219
SARS-CoV-2-31	48.00626	36.83239844	144.623
SARS-CoV-2-32	44.98327	45.1138613	19.48371
SARS-CoV-2-33	70.07409	56.31758199	156.1358
SARS-CoV-2-34	68.86489	74.40275209	54.52269
SARS-CoV-2-35	28.65912	19.88442331	104.0779
SARS-CoV-2-36	36.51889	44.17748621	128.6052
SARS-CoV-2-37	69.46949	81.64394848	184.167
SARS-CoV-2-38	28.35682	28.43786577	144.623
SARS-CoV-2-39	81.25916	130.1535445	118.594
SARS-CoV-2-40	88.21204	73.18397592	56.52489
SARS-CoV-2-41	55.86604	63.23361344	149.6286
SARS-CoV-2-42	113.3029	134.4615149	94.56723
SARS-CoV-2-43	60.70282	73.29865406	57.02553

SARS-CoV-2-44	57.37753	45.88428832	94.06672
SARS-CoV-2-45	42.26258	53.74177421	134.6118
SARS-CoV-2-46	41.65798	47.37170324	81.05221
SARS-CoV-2-47	36.82119	50.50286313	82.05338
SARS-CoV-2-48	61.91201	62.69905569	20.48475
SARS-CoV-2-49	28.35682	19.03539968	57.52605
SARS-CoV-2-50	35.3097	34.17558836	108.5829
SARS-CoV-2-51	101.5132	89.74453259	88.05999
SARS-CoV-2-52	54.95914	59.29075765	87.05894
SARS-CoV-2-53	51.93615	62.65258581	145.1236
SARS-CoV-2-54	42.56488	45.89020524	34.50041
SARS-CoV-2-55	68.56259	56.52414476	38.50482
SARS-CoV-2-56	28.35682	28.99619422	37.50378
SARS-CoV-2-57	46.49476	51.01360323	20.48475
SARS-CoV-2-58	76.72467	63.11128168	113.0879
SARS-CoV-2-59	87.30514	71.06471195	153.1325
SARS-CoV-2-60	31.37981	39.84997011	25.49032
SARS-CoV-2-61	31.98441	36.7260313	54.02217
SARS-CoV-2-62	163.1822	145.6679472	239.2283
SARS-CoV-2-63	51.63385	45.76379168	110.0845
SARS-CoV-2-64	31.07751	40.5622465	0.4624789
SARS-CoV-2-65	33.1936	31.91185133	18.98307
SARS-CoV-2-66	37.12349	34.2460905	57.02553
SARS-CoV-2-67	38.03039	39.98268911	80.55169
SARS-CoV-2-68	150.7879	123.9315838	175.157
SARS-CoV-2-69	43.16947	45.70818748	37.50378
SARS-CoV-2-70	36.82119	39.90919796	150.6297
SARS-CoV-2-71	50.12235	55.32953241	102.0756
SARS-CoV-2-72	66.1442	50.80693544	194.1782
SARS-CoV-2-73	51.02925	56.08994317	113.0879
SARS-CoV-2-74	69.77179	44.09669086	148.1269
SARS-CoV-2-75	128.1155	88.12132122	85.55726
SARS-CoV-2-76	31.98441	85.02409148	112.5874

SARS-CoV-2-77	42.26258	38.19724146	5.468047
SARS-CoV-2-78	41.96028	39.90671689	134.6118
SARS-CoV-2-79	28.65912	29.28410801	-1.539725
SARS-CoV-2-80	41.65798	37.97536878	53.52153
SARS-CoV-2-81	44.37867	37.76643577	87.55946
SARS-CoV-2-82	34.4028	29.75945682	0.9629999
SARS-CoV-2-83	69.46949	73.6814222	122.5985
SARS-CoV-2-84	477.5732	597.8992381	600.1302
SARS-CoV-2-85	345.1662	342.517685	387.3933
SARS-CoV-2-86	421.0433	437.9494649	583.6118
SARS-CoV-2-87	359.6766	341.523034	288.7836
SARS-CoV-2-88	580.3549	569.6223222	487.0042
SARS-CoV-2-89	491.479	483.0877223	465.9808
SARS-CoV-2-90	759.9205	812.7781127	999.5749
SARS-CoV-2-91	306.7743	298.4915217	381.8872
SARS-CoV-2-92	248.4305	235.6958849	292.788
SARS-CoV-2-93	319.4708	319.8755164	332.332
SARS-CoV-2-94	351.8168	350.354742	367.8716
SARS-CoV-2-95	288.334	281.5820718	280.274
SARS-CoV-2-96	259.6156	270.2420956	257.2484
SARS-CoV-2-97	597.2836	681.5567134	599.129
SARS-CoV-2-98	406.8352	360.8253155	365.3688
SARS-CoV-2-99	327.3306	356.0869026	316.3142
SARS-CoV-2-100	264.7547	269.0760229	329.8292
SARS-CoV-2-101	470.0157	384.0174859	516.0366
SARS-CoV-2-102	614.817	551.9465207	739.7857
SARS-CoV-2-103	444.9249	468.1046982	625.6586
Pre-SARS-CoV-2-1	<4.173	0.673568393	-10.04918
Pre-SARS-CoV-2-2	<4.173	0.360092686	-39.08155
Pre-SARS-CoV-2-3	<4.173	0.78715145	39.50598
Pre-SARS-CoV-2-4	<4.173	0.579140984	7.970891
Pre-SARS-CoV-2-5	<4.173	0.552729444	8.471412
Pre-SARS-CoV-2-6	<4.173	0.527459842	-25.56652

Pre-SARS-CoV-2-7	<4.173	0.602107726	36.50262
Pre-SARS-CoV-2-8	<4.173	0.440246082	50.01764
Pre-SARS-CoV-2-9	<4.173	0.757053724	17.48139
Pre-SARS-CoV-2-10	<4.173	0.417723781	24.48928
Pre-SARS-CoV-2-11	<4.173	0.959584601	31.49705
Pre-SARS-CoV-2-12	<4.173	0.505217052	37.50378
Pre-SARS-CoV-2-13	<4.173	0.38753283	-42.08491
Pre-SARS-CoV-2-14	<4.173	0.576967433	-33.07482
Pre-SARS-CoV-2-15	<4.173	0.451723308	-3.041407
Pre-SARS-CoV-2-16	<4.173	0.392535374	37.50378
Pre-SARS-CoV-2-17	<4.173	0.355587757	0.4624789
Pre-SARS-CoV-2-18	<4.173	0.477914272	-12.55202
Pre-SARS-CoV-2-19	<4.173	0.681154453	50.51828
Pre-SARS-CoV-2-20	<4.173	0.632534183	35.50146
Pre-SARS-CoV-2-21	<4.173	0.696621221	18.98307
Pre-SARS-CoV-2-22	<4.173	0.355890996	-10.54982
Pre-SARS-CoV-2-23	<4.173	0.33929511	48.01544
Pre-SARS-CoV-2-24	<4.173	0.565380337	58.52709
Pre-SARS-CoV-2-25	<4.173	0.78609331	52.01985
Pre-SARS-CoV-2-26	<4.173	0.464173989	41.00766
Pre-SARS-CoV-2-27	<4.173	0.424441949	38.0043
Pre-SARS-CoV-2-28	<4.173	0.670700299	15.47918
Pre-SARS-CoV-2-29	<4.173	0.575713085	-40.58323
Pre-SARS-CoV-2-30	<4.173	0.428691823	-18.05811
Pre-SARS-CoV-2-31	<4.173	0.848953983	-28.56977
Pre-SARS-CoV-2-32	<4.173	1.091301495	-1.039204
Pre-SARS-CoV-2-33	<4.173	0.992799949	18.98307
Pre-SARS-CoV-2-34	<4.173	0.848721922	-34.5765
Pre-SARS-CoV-2-35	<4.173	1.153911257	25.49032
Pre-SARS-CoV-2-36	<4.173	0.32559513	-11.05034
Pre-SARS-CoV-2-37	<4.173	1.166829391	20.48475
Pre-SARS-CoV-2-38	<4.173	0.593252395	47.01428
Pre-SARS-CoV-2-39	<4.173	0.88569634	-36.07818

Pre-SARS-CoV-2-40	<4.173	0.585667197	-31.57313
Pre-SARS-CoV-2-41	<4.173	0.501473052	-2.540886
Pre-SARS-CoV-2-42	<4.173	0.545405232	-39.08155
Pre-SARS-CoV-2-43	<4.173	0.702531497	38.0043
Pre-SARS-CoV-2-44	<4.173	0.562220918	8.971933
Pre-SARS-CoV-2-45	<4.173	0.700615595	33.49925
Pre-SARS-CoV-2-46	<4.173	0.547405681	-18.05811
Pre-SARS-CoV-2-47	<4.173	0.639167671	8.471412
Pre-SARS-CoV-2-48	<4.173	0.537001872	27.99316
Pre-SARS-CoV-2-49	<4.173	0.547753297	18.98307
Pre-SARS-CoV-2-50	<4.173	0.487723184	45.51271

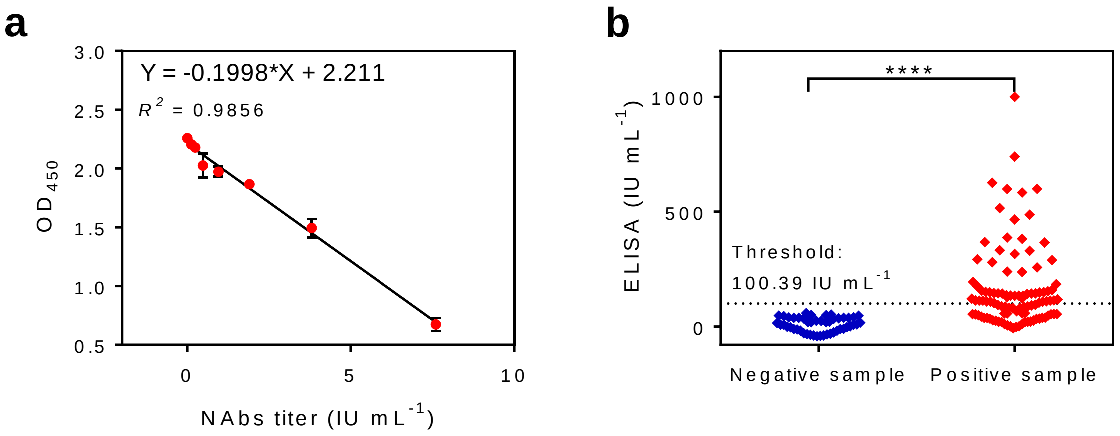

Figure S23. The dose-response curve (a) and detection results (b) of the commercial ELISA kit (the dotted line was LoD of the method).

Performance of the AIE$_{490}$NP-based LFIA

The AIE$_{490}$NP-based LFIA was performed as a typical competitive immunoassay. As shown in Scheme S2, the RBD protein and anti-rabbit IgG were coated on the NC membrane as the test line and control line, respectively. Meanwhile, the AIE$_{490}$NP-ACE2 and AIE$_{490}$NP-rabbit IgG (RIgG) were prepared using the EDC/NHS method and immobilized on the conjugate pad as fluorescence labels. The particle size and zeta potential of AIE$_{490}$NP-ACE2 (381.27 nm and -32.5 mV) and AIE$_{490}$NP-RIgG (340.17 nm and -29.87 mV) were shown in

Figure S23a and S23b, indicating successful modification of ACE2 or RIgG on the surface of $AIE_{490}NP$.

When detecting the serum sample using the $AIE_{490}NP$-based LFIA test strip, the sample buffer containing the serum sample was loaded on the sample pad and then migrated to the conjugate pad followed by NC membrane by capillary action. After the fluorescence labels flowing with the buffer reached the test line, the $AIE_{490}NP$-ACE2 would bound with the RBD protein and exhibited bright fluorescence at the test line, and then the control line exhibited too due to the combination of anti-rabbit IgG with $AIE_{490}NP$-RIgG. Once the serum sample contained anti-SARS-CoV-2 NAbs, the combination of $AIE_{490}NP$-ACE2 with the RBD protein would be restrained and the fluorescence signal of the test line decreased due to less $AIE_{490}NP$-ACE2 on it, which meant the high titer of anti-SARS-CoV-2 NAbs in the serum sample leading weak fluorescence signal on the test line.

The incubation time was optimized by detecting the sample buffer using the strip and monitoring the change trends of the fluorescence signal of the test line (H_T) and control line (H_C) with 5 replicates (Figure S23c and S23d). Comprehensively considering the H_T, H_C, ratio of H_T/H_C, and the CV% of H_T/H_C ratio, the incubation time was optimized to be 12 min. Through detecting a series of calibrators (Figure S23e), the ratio of H_T/H_C exhibited a logit correlation to the NAbs titers of the calibrators with the response curve of logit (Y) = -3.175 * log (X) + 7.024 (R^2 = 0.9577) (Figure S23e and S23f). The logit (Y) was calculated as ln $[(R/R_0)/(1-R/R_0)]$, where R is the H_T/H_C ratio of the calibrator and R_0 is that of the sample buffer.

To evaluate the performance of the $AIE_{490}NP$-based LFIA, 20 negative serum samples and 20 positive serum samples were detected using the test strips. The detection results (Figure S23g) suggested that the $AIE_{490}NP$-based LFIA could effectively distinguish the positive and negative serum samples and the LoD of the method was calculated to be 11.06 IU mL^{-1}. And the $AIE_{490}NP$-based LFIA results were significantly correlated to the pVNT method (R^2 = 0.9581, P<0.0001) (Figure S23h).

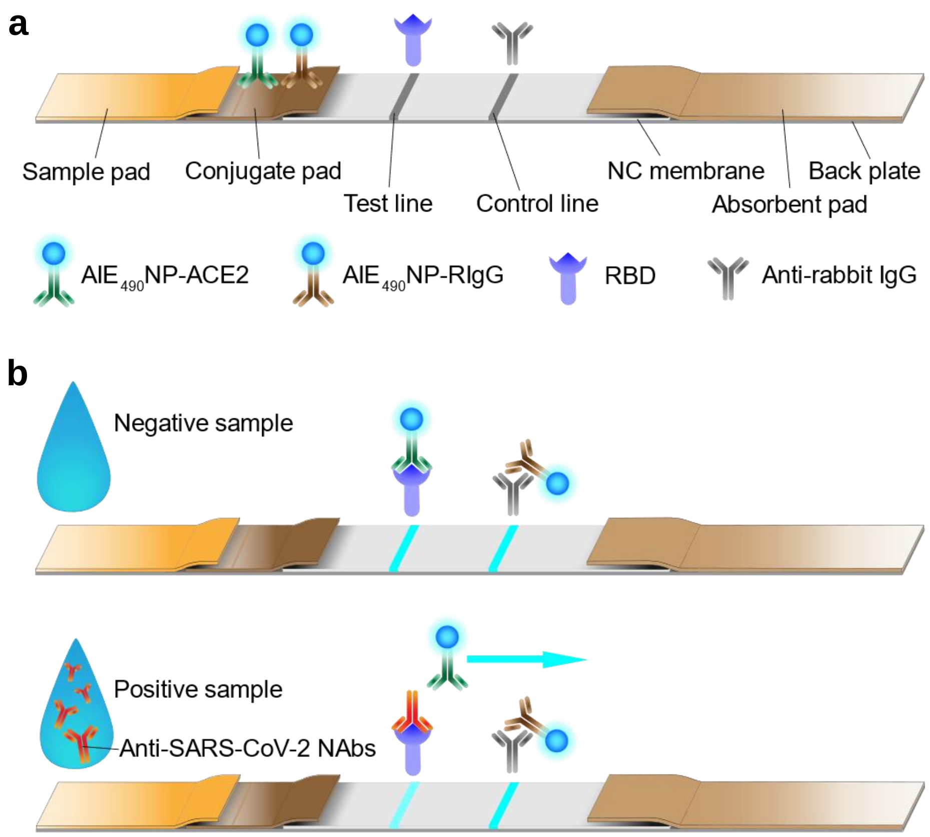

Scheme S2. (a) The structure of the established $AIE_{490}NP$-based LFIA test strip product. (b) Schematic of the established $AIE_{490}NP$-based LFIA test strip for detection of anti-SARS-CoV-2 NAbs in human serum samples.

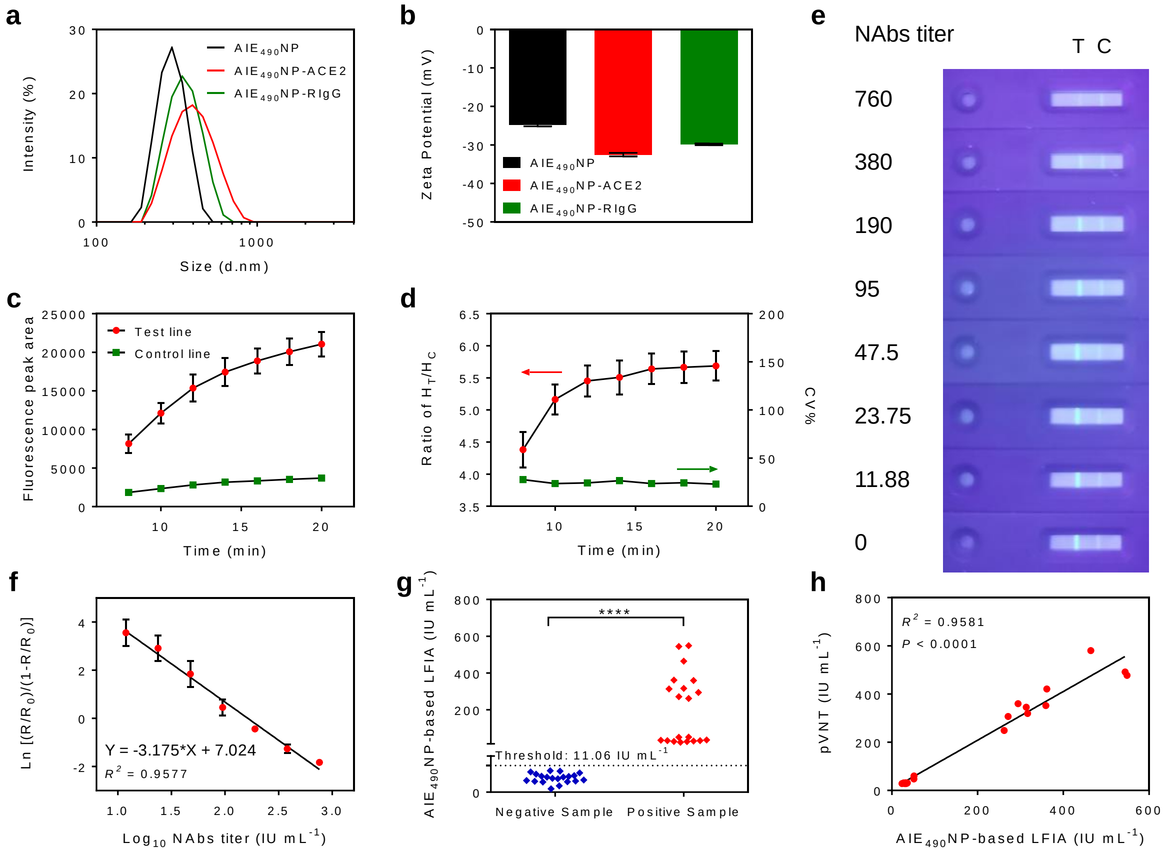

Figure S24. The particle size (a) and zeta potential (b) of $AIE_{490}NP$, $AIE_{490}NP$-ACE2, and $AIE_{490}NP$-RIgG. (c) The change trends of H_T and H_C along with the incubation time. (d) The change trends of the H_T/H_C ratio along with the incubation time. (e) The pictures of test strips used for detecting the calibrators with NAbs titers of 760, 380, 190, 95, 47.5, 23.75, and 11.88 IU mL^{-1}. (f) The dose-response curve of the $AIE_{490}NP$-based LFIA. (g) The detection results of the 20 negative and 20 positive samples obtained using the $AIE_{490}NP$-based LFIA (the dotted line was the LoD of the method). (h) The comparison between the $AIE_{490}NP$-based LFIA and pVNT in detecting the 20 positive serum samples.

Table S6. The anti-SARS-CoV-2 NAbs titers (wild-type strain and Delta-variant strain) of serum samples (pVNT).

Sample NO.	pVNT Delta-variant strain (IU mL^{-1})	FQ-LFIA (IU mL^{-1})
SARS-CoV-2-1	<2.941	29
SARS-CoV-2-2	23	90
SARS-CoV-2-3	<2.941	29
SARS-CoV-2-4	4	33
SARS-CoV-2-5	21	32
SARS-CoV-2-6	39	24
SARS-CoV-2-7	23	27

SARS-CoV-2-8	7	28
SARS-CoV-2-9	10	40
SARS-CoV-2-10	29	43
SARS-CoV-2-16	31	65
SARS-CoV-2-17	9	38
SARS-CoV-2-18	14	41
SARS-CoV-2-19	21	64
SARS-CoV-2-20	14	32
SARS-CoV-2-21	19	32
SARS-CoV-2-22	20	104
SARS-CoV-2-23	9	50
SARS-CoV-2-24	36	59
SARS-CoV-2-25	6	36
SARS-CoV-2-26	18	54
SARS-CoV-2-27	14	53
SARS-CoV-2-28	19	59
SARS-CoV-2-29	6	41
SARS-CoV-2-30	12	46
SARS-CoV-2-31	54	37
SARS-CoV-2-32	7	45
SARS-CoV-2-33	22	56
SARS-CoV-2-34	49	74
SARS-CoV-2-35	6	20
SARS-CoV-2-36	6	44
SARS-CoV-2-37	27	82
SARS-CoV-2-38	7	28
SARS-CoV-2-39	6	130
SARS-CoV-2-40	53	73
SARS-CoV-2-41	23	63
SARS-CoV-2-42	31	134
SARS-CoV-2-66	8	34
SARS-CoV-2-67	16	40
SARS-CoV-2-68	240	124
SARS-CoV-2-69	20	46
SARS-CoV-2-70	16	40
SARS-CoV-2-71	64	55
SARS-CoV-2-72	40	51
SARS-CoV-2-73	9	56
SARS-CoV-2-74	21	44
SARS-CoV-2-75	53	88
SARS-CoV-2-85	287	343
SARS-CoV-2-86	433	438
SARS-CoV-2-89	317	483
SARS-CoV-2-90	488	813
SARS-CoV-2-91	205	298
SARS-CoV-2-100	216	269

SARS-CoV-2-101	71	384
SARS-CoV-2-102	441	552
SARS-CoV-2-103	326	468

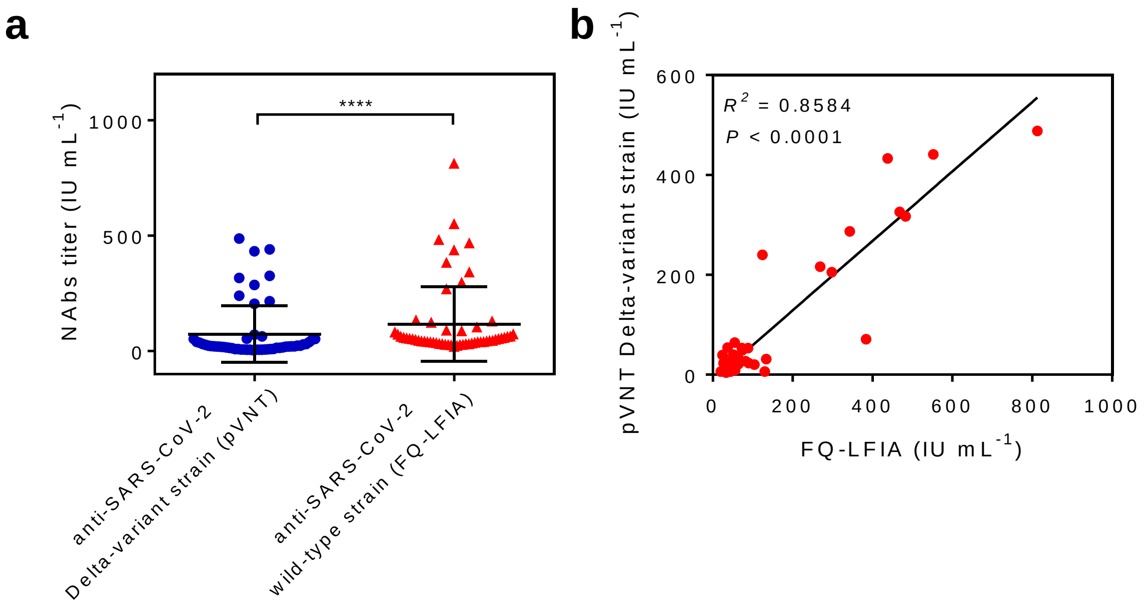

Figure S25. (a) The anti-SARS-CoV-2 Delta-variant strain NAbs titers (pVNT) and anti-SARS-CoV-2 wild-type strain NAbs titers (FQ-LFIA). (b) Comparison between anti-SARS-CoV-2 Delta-variant strain NAbs titers (pVNT) and anti-SARS-CoV-2 wild-type strain NAbs titers (FQ-LFIA) of 56 serum samples.
